# Supplementary material for: Half-metallic carbon nitride nanosheets with micro grid mode resonance structure for efficient photocatalytic hydrogen evolution
Source: Nat Commun. 2018 Aug 22;9:3366. doi: 10.1038/s41467-018-05590-x (PMC6105617; doi:10.1038/s41467-018-05590-x)
Supplement: Supplementary file 1 — Supplementary Information [file 41467_2018_5590_MOESM1_ESM.pdf]

Supplementary Information for Manuscript

# Half-Metallic Carbon Nitride Nanosheets with Micro Grid Mode Resonance Structure for Efficient Photocatalytic Hydrogen Evolution

**Zhou et al.**

# Supplementary Figures

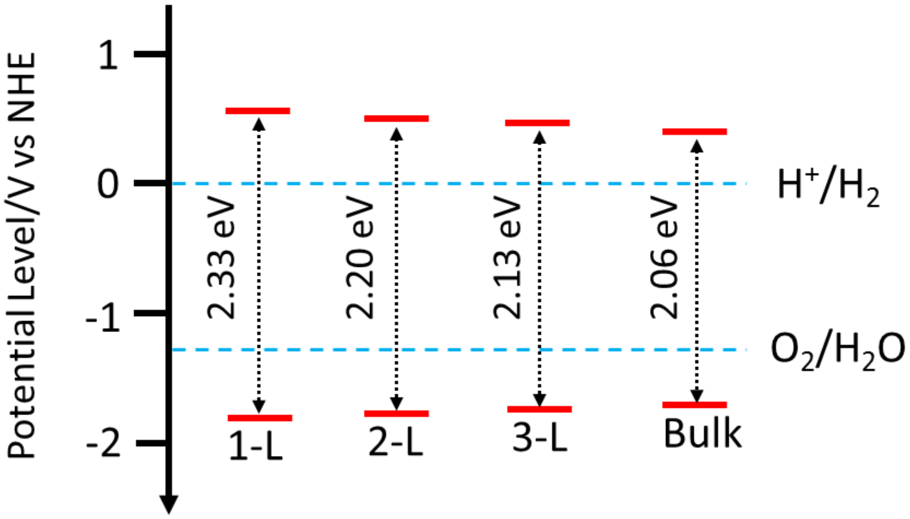


**Supplementary Figure 1. Band-edge positions of the layered hm-C(CN)$_3$ relative to the vacuum level.**

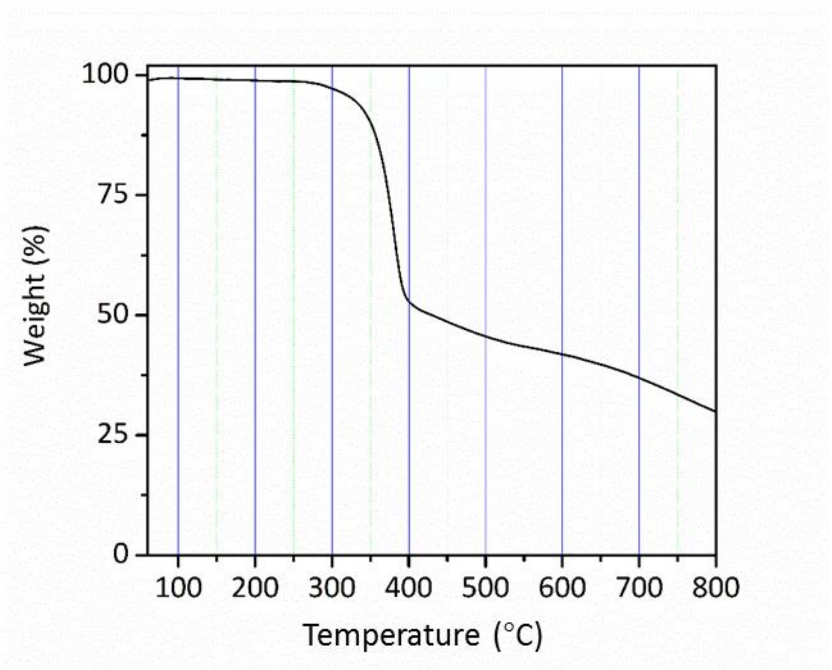


**Supplementary Figure 2. Scanning thermal gravimetric analysis (TGA) of the ionic liquid precursor for hm-$C(CN)_3$ sample.**

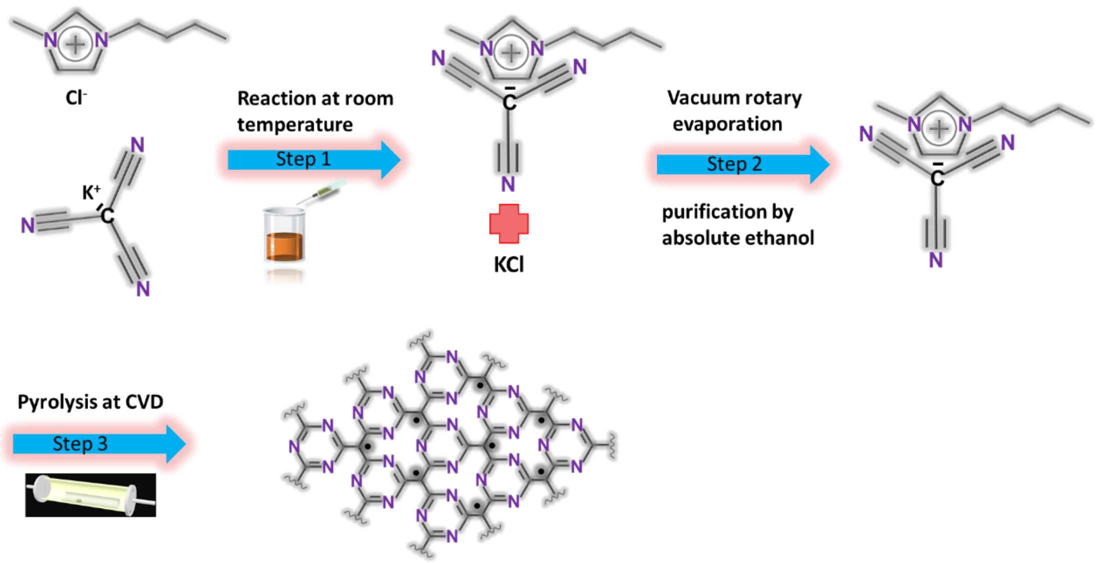


**Supplementary Figure 3. The schematic representation of preparation route for hm-$C(CN)_3$ sample.**

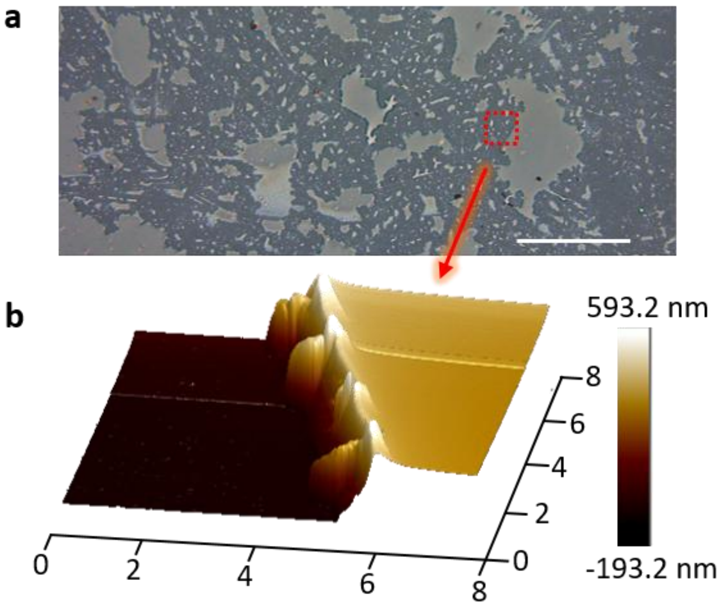


**Supplementary Figure 4. The size of bulk hm-$C(CN)_3$.**

**a** Optical images and **b** AFM of the bulk hm-$C(CN)_3$ samples.

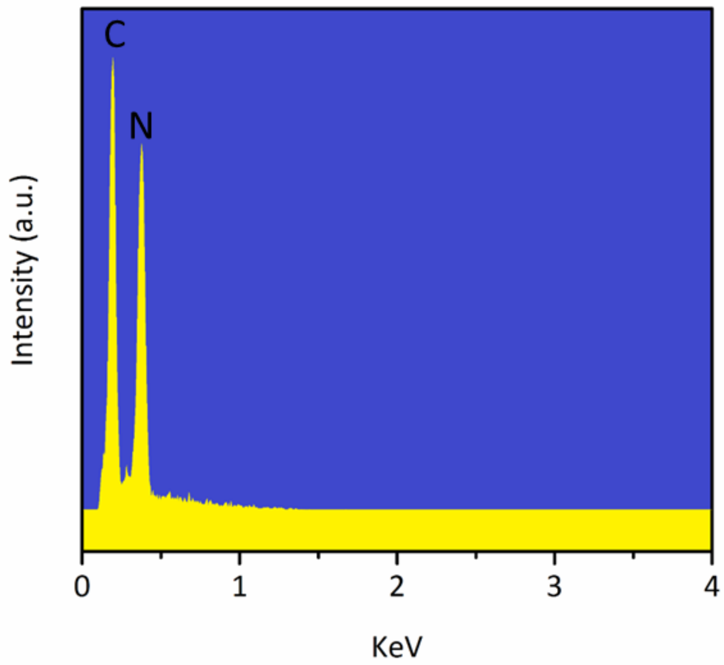


**Supplementary Figure 5. EDX spectrum of the hm-$C(CN)_3$ nanosheets.**

No noticeable impurity elements are introduced unintentionally in the prepared process.

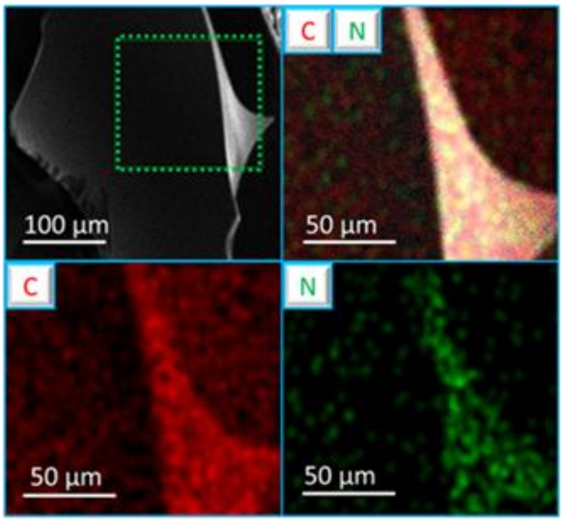


**Supplementary Figure 6. EDX mapping of the bulk hm-$C(CN)_3$ materials.**

The C and N elements are uniformly distributed in the mapping image.

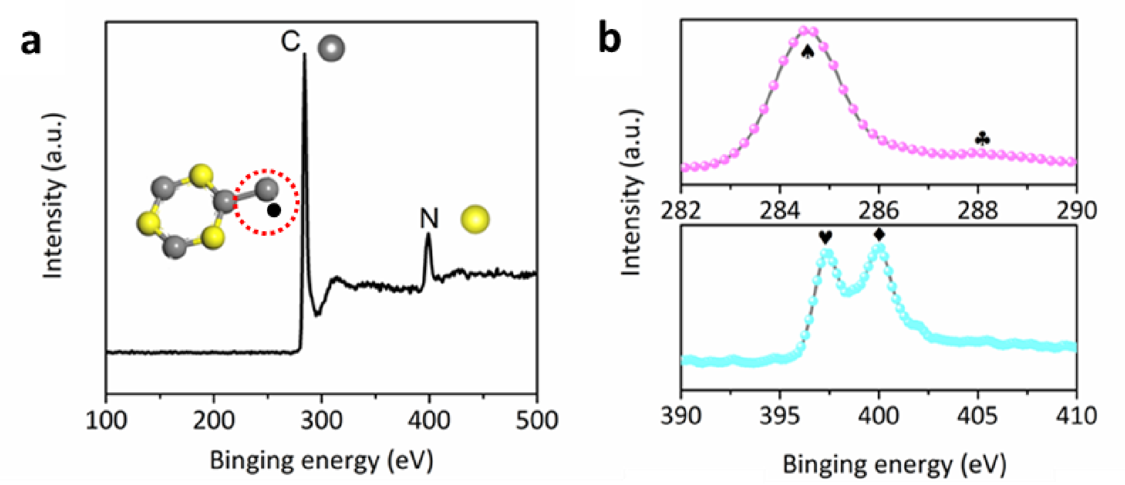


**Supplementary Figure 7. XPS spectra of the hm-C(CN)$_3$.**

XPS spectra for **a** survey; **b** C 1S (top) and N 1S (bottom) of the bulk hm-C(CN)$_3$ materials. The X-ray photoelectron spectra (XPS) further confirms the element composition (only C and N) and the successful preparation of hm-C(CN)$_3$ (Supplementary Figure 7a). In the C 1s spectrum of hm-C(CN)$_3$, two main peaks are observed at the binding energies of 284.8 (marked by ♠) and 288.1 eV (marked by ♣) (top of the Supplementary Figure 7b), which are assigned to sp$^3$-coordinated carbon bond (C−C) and sp$^2$-bonded carbon (C-N), respectively[2,3]. The N 1s XPS spectrum exhibits two peaks at 398.6 (marked by ♥) and 400.5 eV (marked by ♦) (bottom of the Supplementary Figure 7b), which are attributed to the aromatic N (C−N=C) and aliphatic (C=N), respectively.

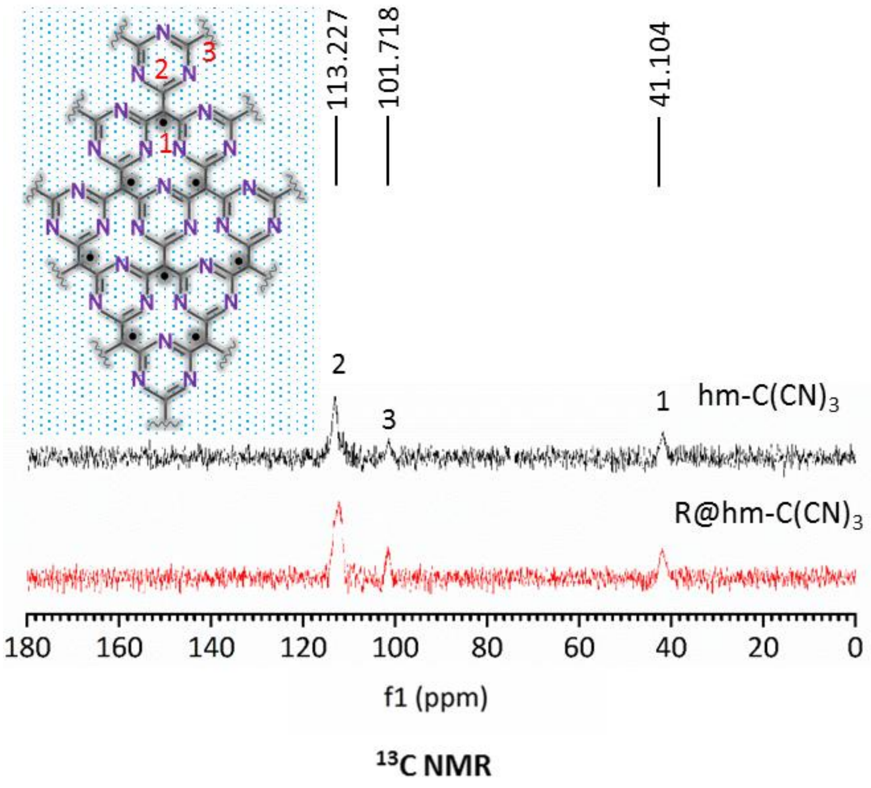


**Supplementary Figure 8.** $^{13}$**C NMR spectra of hm-C(CN)$_3$ bulk sample and R@hm-C(CN)$_3$ nanosheets.**

The weak peak 3 of bulk hm-C(CN)$_3$ gets slightly stronger in nanosheets due to their broken symmetry induced by AAM template.

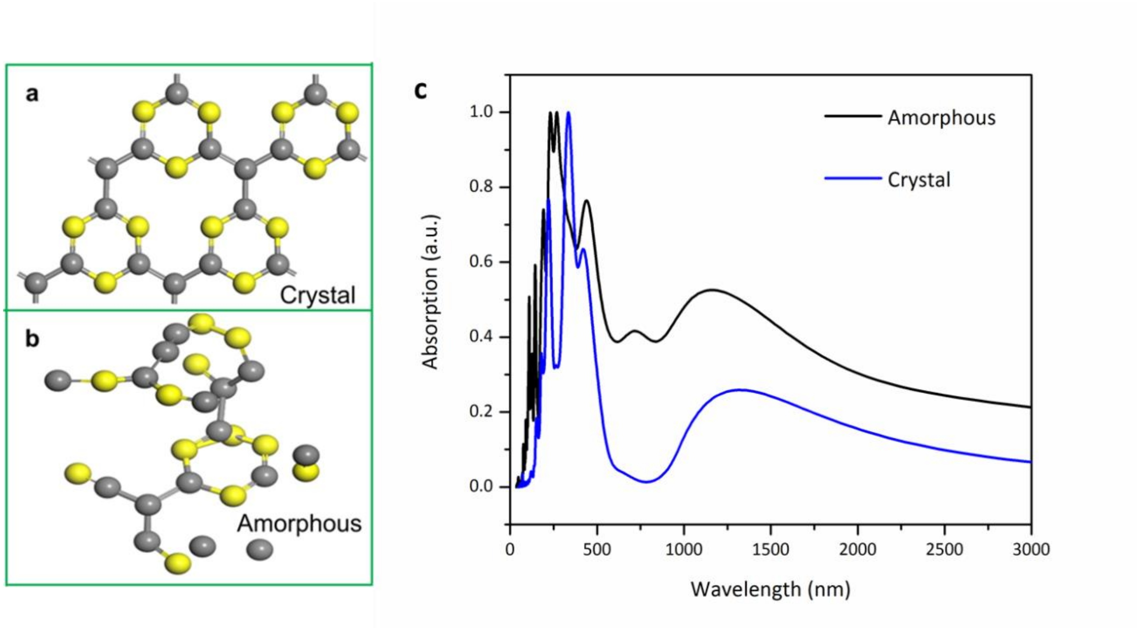


**Supplementary Figure 9. The optical absorption of crystalline and amorphous hm-C(CN)$_3$.**

The atomic model of **a** crystalline and **b** amorphous hm-C(CN)$_3$. **c** The absorption spectra for different atomic models.

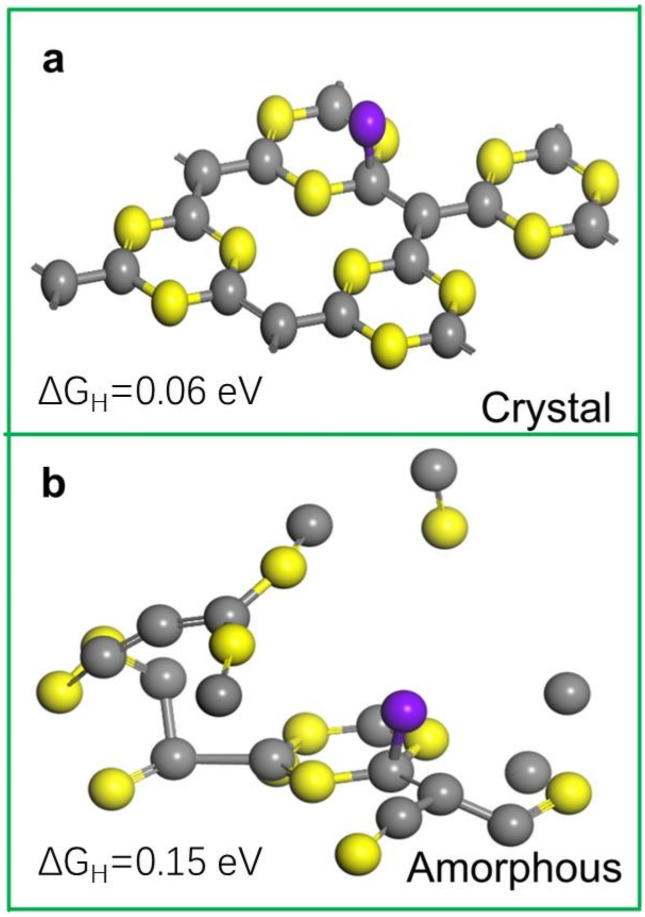


**Supplementary Figure 10. The free-energy and schematic structural of crystalline and amorphous hm-$C(CN)_3$.**

**a** Free-energy and schematic structural representations for hydrogen ($H^*$) adsorption at the C site on the surface of crystalline hm-$C(CN)_3$ and **b** the amorphous hm-$C(CN)_3$. The carbon atoms are grey, nitrogen atoms are yellow and the hydrogen atoms are purple.

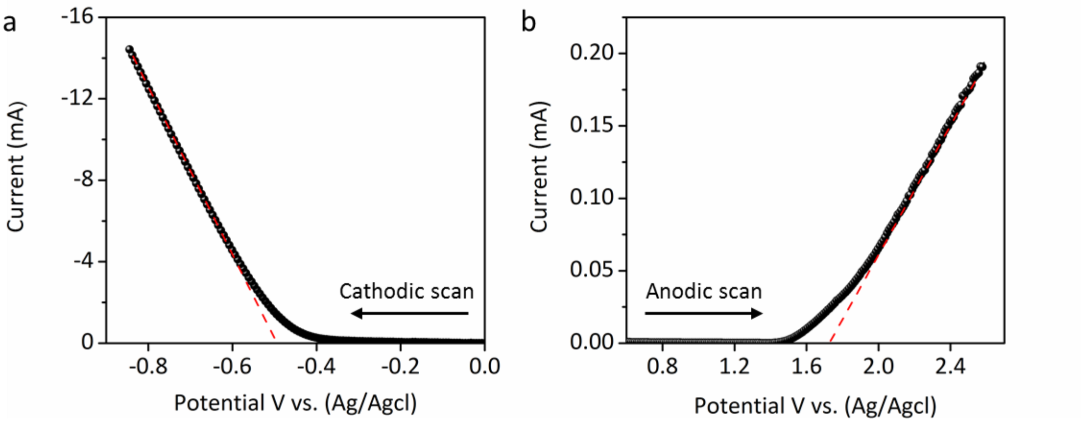


**Supplementary Figure 11. The band-gap energy measurement.**

**a** Cathodic and **b** anodic scans for determining the CB and VB energy levels[4] of hm-$C(CN)_3$ at 5 mV $s^{-1}$. Applying potentials above the CB to form an accumulation layer, or below the VB to form inversion layers, can lead to abrupt emergence of cathodic and anodic currents, respectively. The band-gap energy of hm-$C(CN)_3$ was determined as 2.19 eV, in agreement with the value obtained from the state-of-the-art hybrid functional (HSE06) calculations.

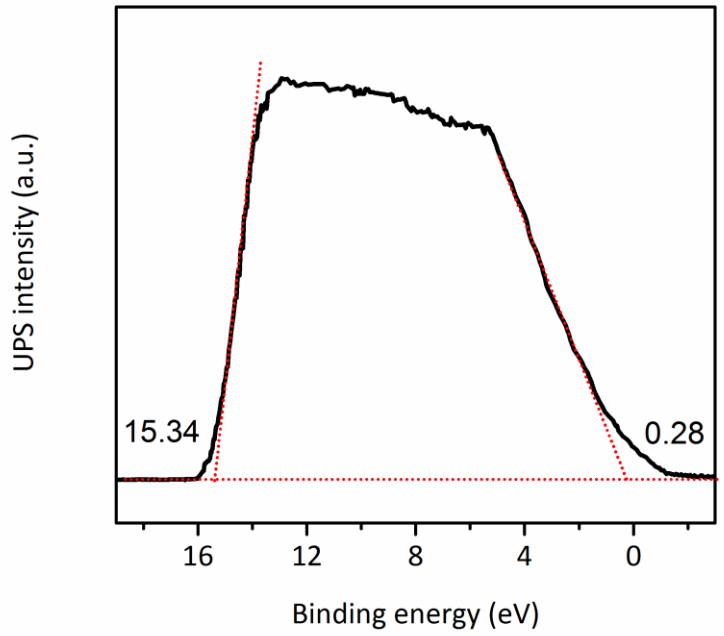


**Supplementary Figure 12. UPS spectra of hm-C(CN)$_3$ nanosheets (black curve)**. The dashed red lines mark the baseline and the tangents of the curve. The intersections of the tangents with the baseline give the edges of the UPS spectra from which the UPS width is determined.

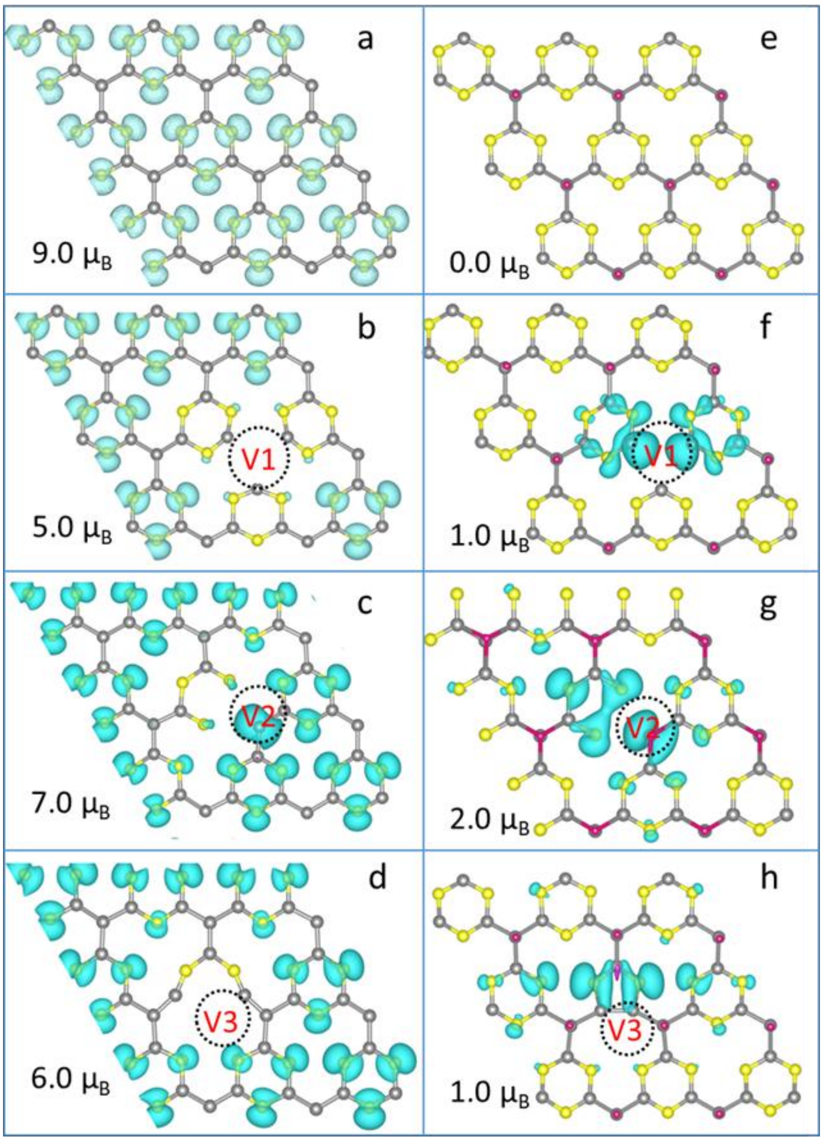


**Supplementary Figure 13. Calculated spin densities of hm-$C(CN)_3$ monolayer with different defects before a-d and after e-h hydrogenation treatment**.

Gray, yellow and pink balls stand for C, N and H atoms, respectively. The spin charge density between spin-up and spin-down ($\rho=\rho_{\downarrow}-\rho_{\uparrow}$) is symmetrically distributed in neighboring N atoms (yellow atom), as shown in Supplementary Figure 13a. This is because the existing radical C sites (targeted by pink H atoms) can inject holes into this structure, and superfluous charges are redistributed in nearby N atoms. The distribution of spin polarization can be affected by different defects, as shown in Supplementary Figures 13b-d, leading to decrease in magnetic moments. After hydrogenation into those structures, the radical C sites are saturated by H atoms (pink atoms) to eliminate superfluous charges, which makes spin polarization and magnetic

moments disappear simultaneously (Supplementary Figure 13e). However, spin polarizations are completely unaffected at defect vicinity (marked by circles) (Supplementary Figures 13f-h). These results demonstrate that the spin polarizations induced by radical C sites are strongly associated with hydrogenation.

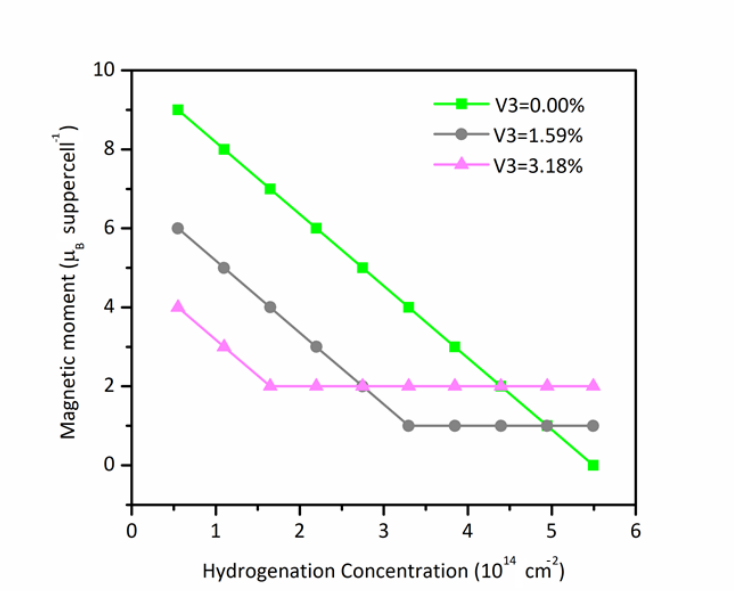


**Supplementary Figure 14. Calculated magnetic moment as a function of hydrogenation concentration in hm-C(CN)$_3$ monolayer with different V3 defect concentrations.**

To more intuitively display contribution of hydrogenation, the magnetic moments of hm-C(CN)$_3$ monolayer with different number of V3 defects as functions of hydrogenation concentration are calculated and compared in Supplementary Figure 14. We found that the magnetic moments of hm-C(CN)$_3$ monolayer without defects drop linearly with increasing hydrogenation concentration, and finally disappear completely, which is quite in agreement with calculated results in Supplementary Figures 13a and 13e. However, when some V3 defects with the lowest formation energy are introduced intentionally, the original magnetic moment values are depressed, which is not benefit for half-metallic observation in spin resolved photoemission spectra. More importantly, the hydrogenation cannot make magnetism originated from defects be eliminated completely. Note that this behavior becomes more remarkable especially for higher defect concentration. This evidences that the magnetic origin can be identified via hydrogenation treatment.

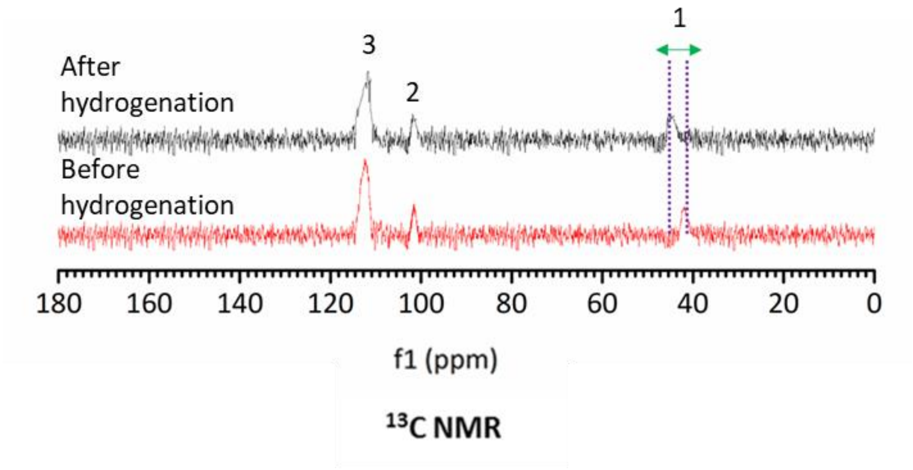


**Supplementary Figure 15. $^{13}C$ NMR spectra of R@hm-$C(CN)_3$ nanosheets before and after hydrogenation.**

To confirm the hydrogenation process, the $^{13}C$ NMR spectra of hm-$C(CN)_3$ sample before and after hydrogenation were measured and shown in Supplementary Figure 15. It is important to note that only three typical peaks are observed in consistence with structural symmetry, which also indicates that no obvious defected structures exist in our samples. This is because the hm-$C(CN)_3$ structure will be destructed, and some new C sites will be visible in NMR spectra, if some defects are introduced unintentionally. In addition, the peak 1 originated from radical C sites slightly shifts after hydrogenation treatment, further confirming that hydrogen deactivation occurs at radical C sites and no additional defect structures are introduced.

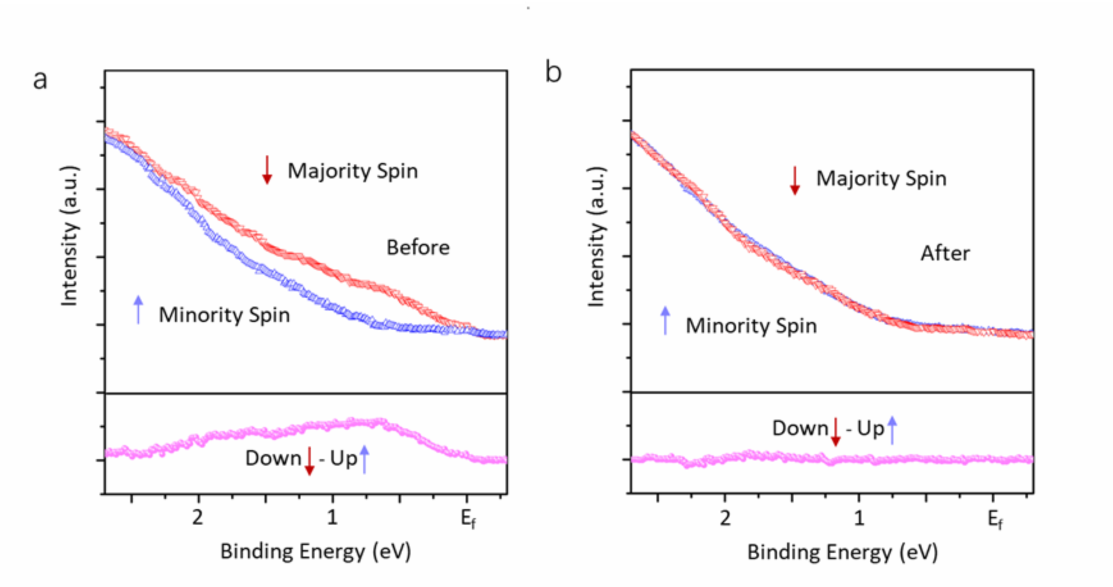


**Supplementary Figure 16. Spin-resolved photoemission spectra near the Fermi energy ($E_F$) of hm-$C(CN)_3$ sample before a and after b hydrogenation at 300 K.** The photon energy was set at hν=40 eV. The bottom panel shows the difference spectrum between the majority-spin and minority-spin spectra. The most striking observation is the half-metallic feature. The spectrum for the majority spin extends up to $E_F$ and shows the metallic Fermi cut-off, while that for the minority spin decreases rapidly at binding energy of ~0.6 eV and the spectral weight disappears very near to $E_F$, reflecting the insulating gap. Previous theoretical calculations disclose that introduction of radical C1 site will inject a hole into original graphitic $C_3N_4$ structure (Ref. 11), which makes spin polarization occur at neighboring N atoms. Therefore, the minority-spin states still show small spectral weight in higher-binding-energy region where the C *p* orbits (C1 site, Figure 6a) are fully occupied by both spins. The *p* orbital electrons of N atoms (N3, N5 and N7 site, Figure 6a) are spin-polarized, leading to ferromagnetic behavior. The spin density of N *p* orbital can be obtained by subtracting the minority-spin spectrum from majority-spin spectrum. The difference spectrum presented in bottom panel of Supplementary Figure 16 shows the metallic Fermi cut-off at $E_F$ and a peak feature around binding energy of 0.75 eV, which can be interpreted as the spin splitting induced by $sp^2$ hybridization. The calculation in Supplementary Table 1 and Supplementary Figures 13-15 imply that magnetic origin can be identified via hydrogenation treatment. This is because that the hydrogenation

cannot make magnetism originated form defects be eliminated completely, but the spin polarization induced by radical C sites can be removed adequately. Subsequently, we experimentally distinguish the physical mechanism of spin resolved photoemission spectra via hydrogenation treatment.

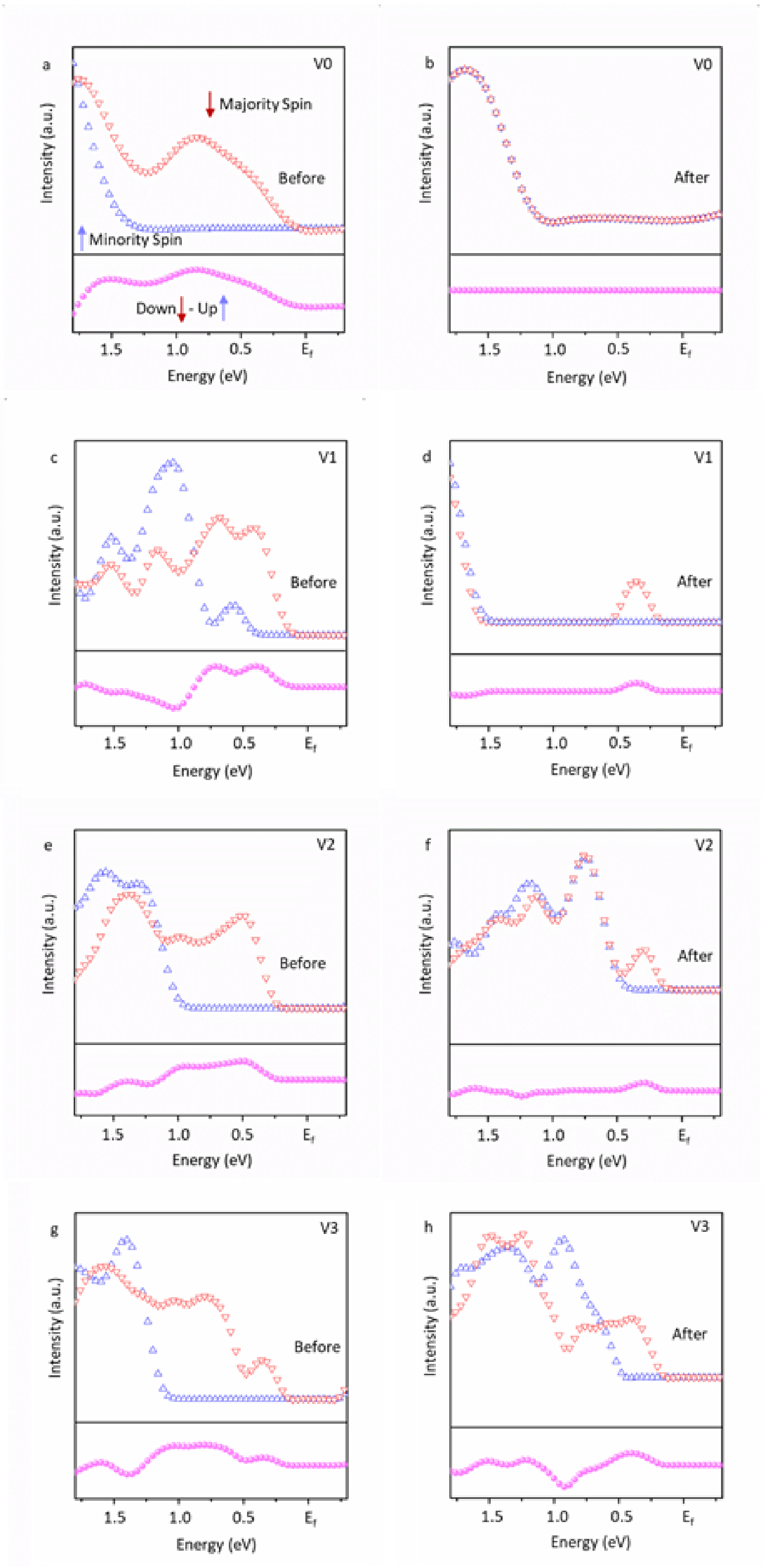


**Supplementary Figure 17. Calculated spin-resolved DOSs and the difference for hm-C(CN)$_3$ monolayer with (V1, V2, V3) and without (V0) defects before a, c, e, g and after b, d, f, h hydrogenation.**

To further confirm our assertions, the spin-resolved DOS for hm-$C(CN)_3$ monolayer with different defects were calculated and shown in Supplementary Figure 17. The results indicate that the spin splitting of DOS can be affected by defects, resulting in changes in spin DOS spectra [DOS difference between majority-spin (down) and minority-spin (up)]. The notable difference between experimental PES spectra and calculated spin-resolved DOS in hm-$C(CN)_3$ monolayer with different defects further evidences that the difference in PES spectra does not result from defects. It is important to note that calculated DOS for pristine hm-$C(CN)_3$ is much similar to experimental PES spectra, as shown in Supplementary Figure 17a, although the spectral difference between majority and minority spins is not so large as the spin resolved DOS by DFT.

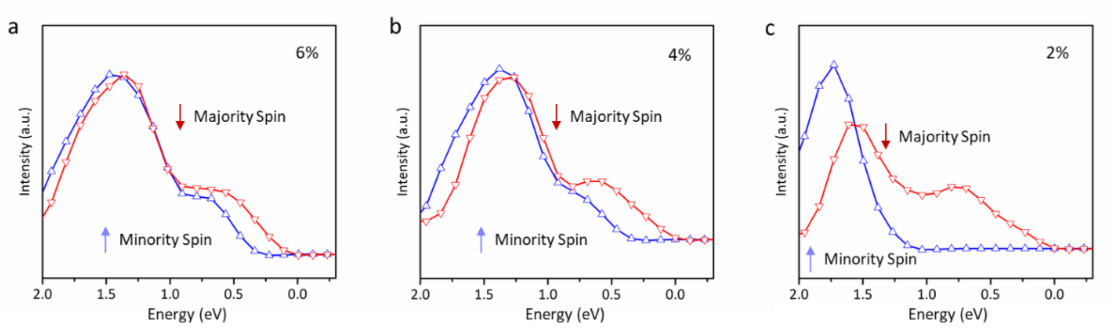


**Supplementary Figure 18. Calculated spin-resolved DOS for hm-C(CN)$_3$ monolayer with different deformation a (6%), b (4%), c (2%)**.

To find the fundamental causes that underlie the spectral differences between PES spectra and spin-resolved DOSs, the polymerization process and XRD patterns of our samples should be discussed in detail. As we all known, experimental samples cannot be as perfect as theoretical simulation, and lattice distortion and deformation cannot be avoided in sample preparation, especially for two dimensional materials [such $MoS_2$ or Graphene[5-7]]. In addition, the broad XRD peak implies that deformation induced by residual strain also takes place in our samples. Considering this physical truth, spin-resolved DOS of hm-C(CN)$_3$ monolayer with different deformations were calculated and shown in Supplementary Figure 18. The calculated results show that the spin splitting in DOS decreases with increasing structural deformation and finally tends to measured PES spectra. This is because spin polarization can be regulated by structural deformation [see our previous report, Ref. 13]. In addition, the stimulated XRD patterns in Supplementary Figure 19 indicate that the broad XRD peak can be assigned to a combination of contributions from hm-C(CN)$_3$ nanosheets with and without deformation. In this context, the structural deformation induced by residual strain can modify the PES feature to a certain extent. Taking this factor into consideration, the differences between experimental PES spectra and DFT predicated DOS can be satisfactorily explained.

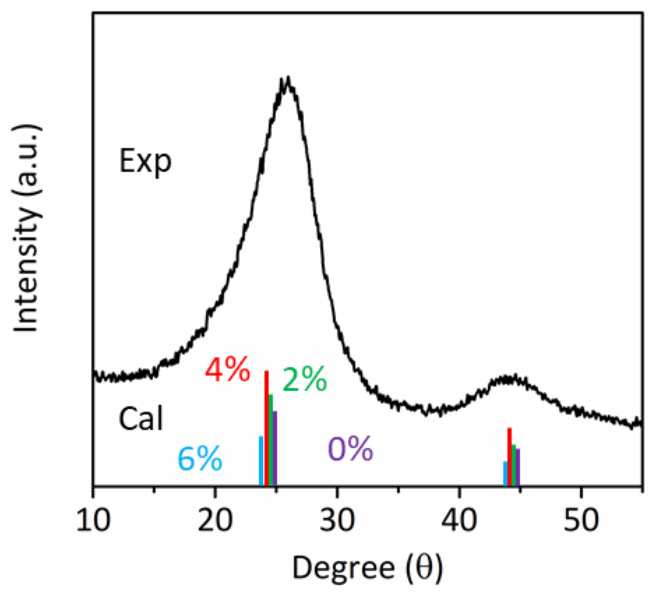


**Supplementary Figure 19. The experimental and the theoretical XRD patterns with different deformations.**

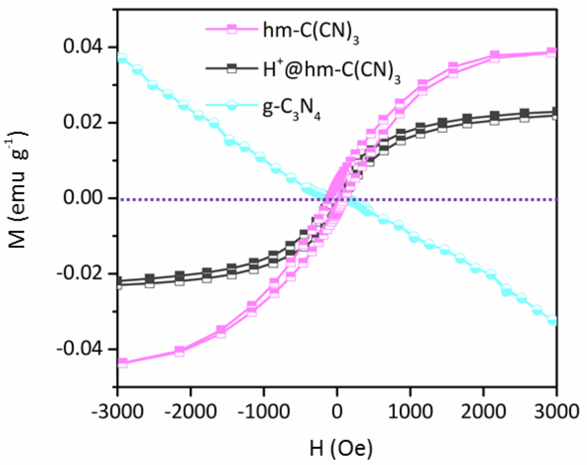


**Supplementary Figure 20. Room temperature magnetic hysteresis loops of the hm-C(CN)$_3$ nanosheets with different post-processing methods and the g-C$_3$N$_4$ as a reference.**

To obtain insights into the magnetic origin of hm-C(CN)$_3$ nanosheets, magnetization as function of magnetic field (M-H) is studied on superconducting quantum interference device (SQUID) for different samples. As shown in Supplementary Figure 20, the nonlinear hysteresis loop curve suggests that hm-C(CN)$_3$ nanosheets are ferromagnetic at room temperature with nozero residual magnetization and coercivity (marked by pink line), while the g-C$_3$N$_4$ displays obvious diamagnetic characteristics. After hm-C(CN)$_3$ nanosheets soaked in dilute hydrochloric acid solutions for 2 hours, the saturation magnetization decreases from 0.038 to 0.02 emu/g but the coercive field **(**$H_c$ =152 Oe) remains unchanged, because the exposed radical C sites are deactivated by adsorptive $H^+$, indicating that the ferromagnetism in hm-C(CN)$_3$ nanosheets is strongly related to the radical C sites. This magnetic behavior is different from the reported[8,9] ferromagnetism in g-C$_3$N$_4$, which is attributed to introduced defects (0.008 emu/g) or external ions (0.004 emu/g).

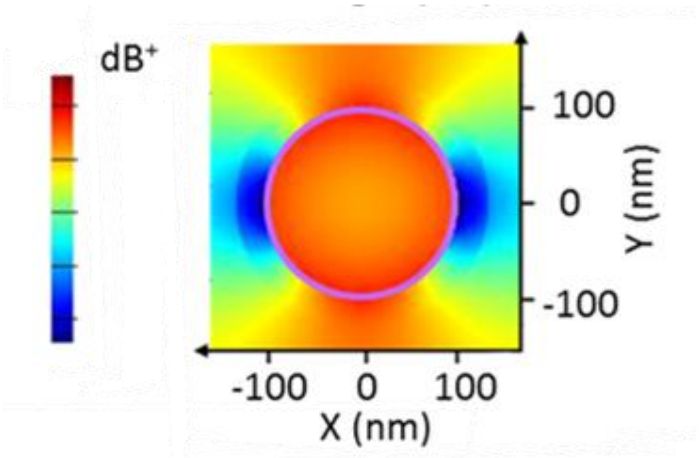


**Supplementary Figure 21. Top-view of the simulated electric field distribution of MG@hm-$C(CN)_3$.**

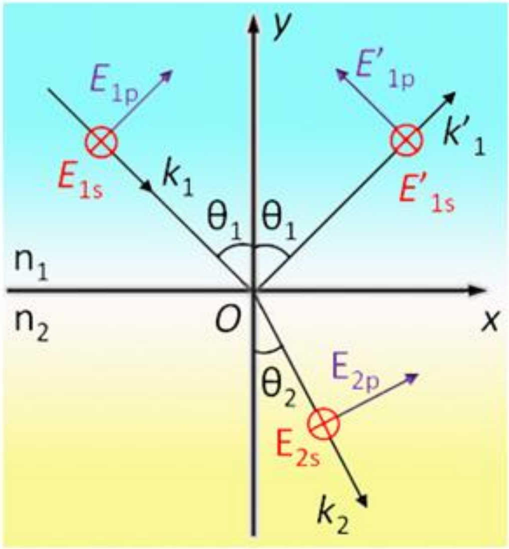


**Supplementary Figure 22. P wave and S wave component of electric field.**

One part parallel to the incident surface is called the P wave (marked by purple arrows: E1p) and the other part perpendicular to the incident surface is called the S wave (marked by red circles: E1s). Ep and Es are the two mutually perpendicular components of Electric vector.

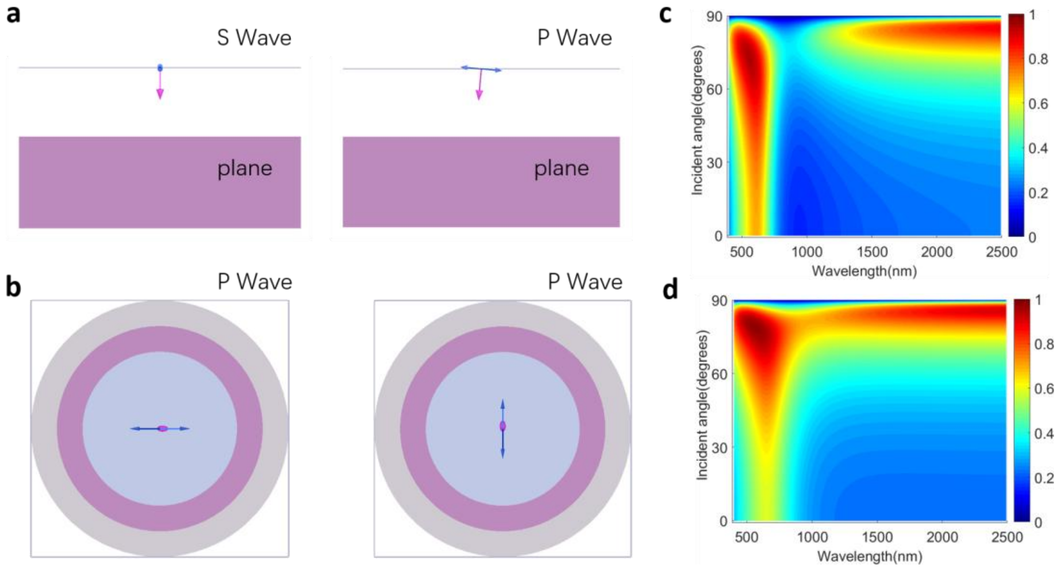


**Supplementary Figure 23. Difference of absorption coefficient between P wave and S wave.**

The spatial position relationship between electric vector and **a** the plane material and **b** the cylindrical structural material. Absorption zones of P-wave versus wavelength and incident angle for smooth **c** and rough **d** inner walls in nanotube arrays. For the plane material, when the natural light is incident or obliquely incident, the component of the electrical vector perpendicular to the surface of the material is P wave, and the component parallel to the surface of the material is S wave. Therefore, for the planar material, the absorption characteristics of natural light are the superposition of S wave and P Wave. However, for the columnar structure material, the transverse component of the electrical vector is always perpendicular to the surface of the material, and its absorption characteristics are mainly determined by the P wave. Generally speaking, both ways of reducing reflections and increasing effective optical path length of an incident light ray are utilized to increase light absorption. Here, in the fabrication process, hm-$C(CN)_3$ nanosheets are randomly incorporated into AAM to construct cylindrical resonators (void resonators), of which the inner walls are rough. Then, this structure can be simply regard as light scatters off nanoscale voids within a high-index dielectric or high-index dielectric surrounded by a low-index embedding medium, typically air or water.

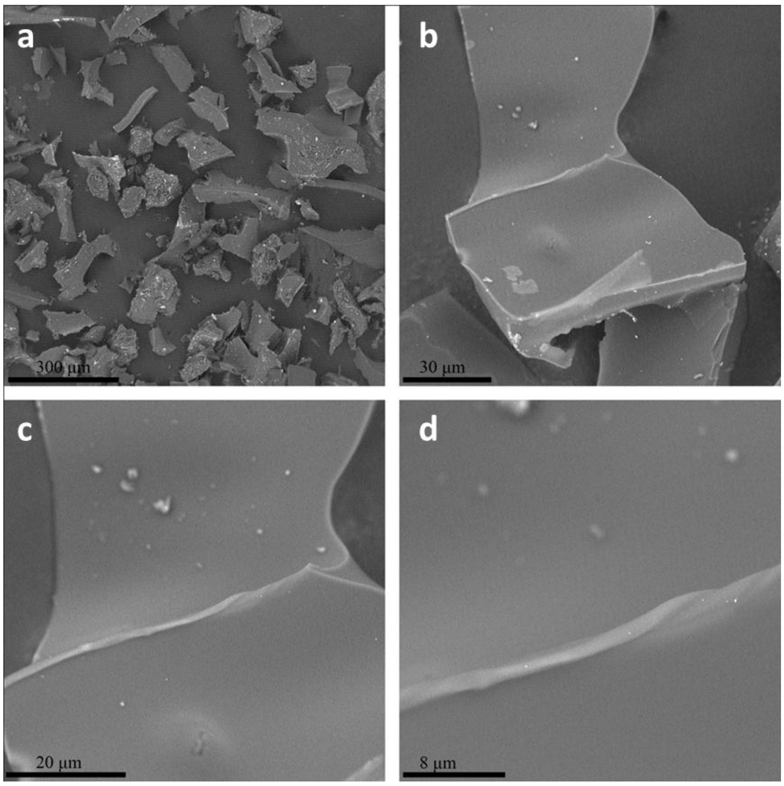


**Supplementary Figure 24. SEM image of hm-$C(CN)_3$.**

**a** SEM image of the synthesized bulk hm-$C(CN)_3$ sheets. **b**, **c** and **d** the applied SEM images of the synthesized bulk hm-$C(CN)_3$ sheets showing the size of ~200 μm.

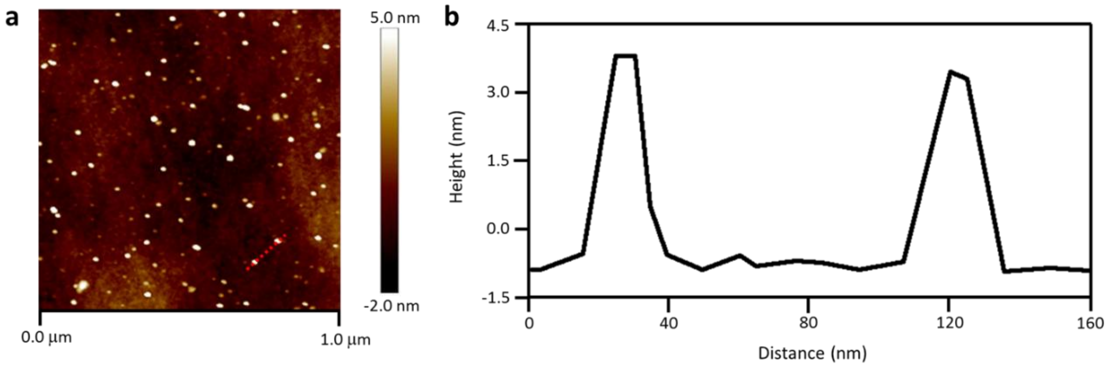


**Supplementary Figure 25. The size of R@hm-C(CN)$_3$ nanosheets**.

**a** AFM image of the synthetized R@hm-C(CN)$_3$ nanosheets. **b** The corresponding height profile of two random nanoparticles from the AFM image. Height profile along the red dotted line in Supplementary Figure 25a indicating the layer thickness of ~ 4.5 nm.

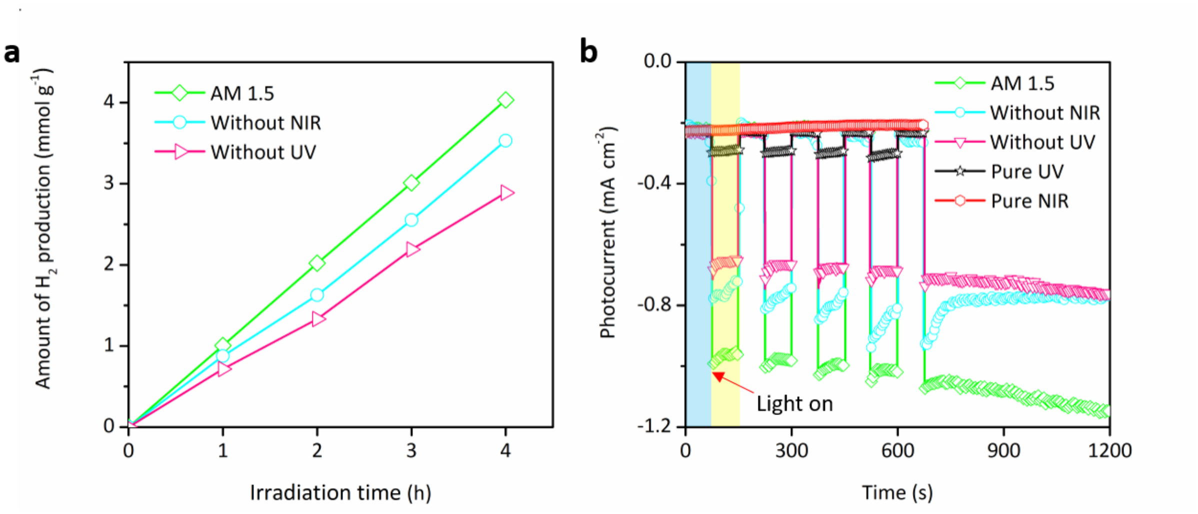


**Supplementary Figure 26. Photocatalytic HER performace of hm-C(CN)$_3$ nanosheets.**

**a** Photocatalytic $H_2$ evolution from water under different irradiation lights on MG@hm-C(CN)$_3$. **b** Amperometric I-t curves of hm-C(CN)$_3$ photocathode at the potential of -0.2 V (vs Ag/AgCl) under the chopped on-off cycles of different light sources: the white lights with and without NIR/UV component, and pure NIR/UV light.

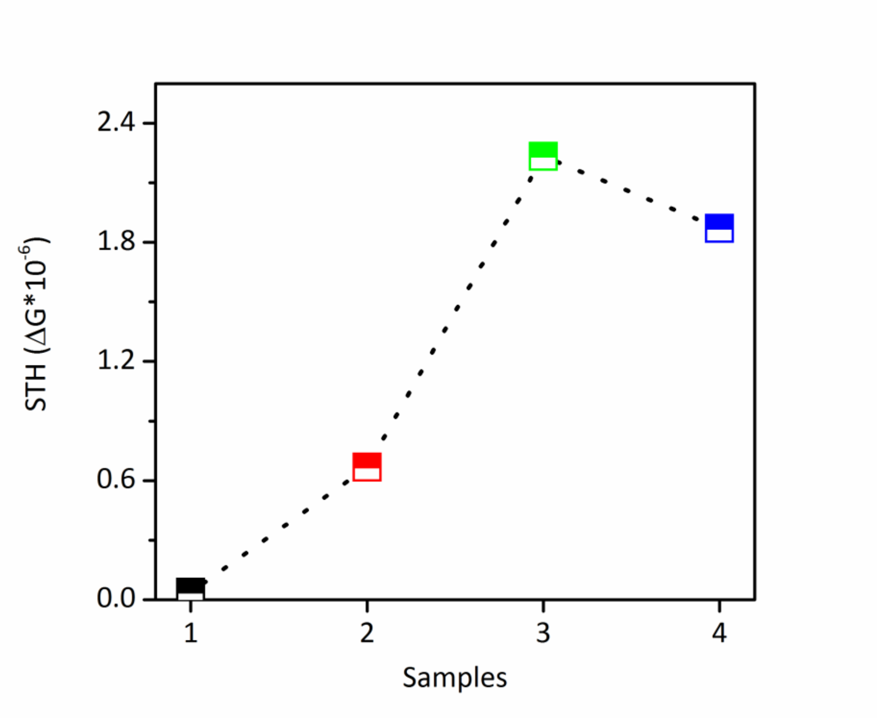


**Supplementary Figure 27. The STH efficiencies for different samples.**

The highest STH (the ratio of the chemical energy in the generated hydrogen to the incoming luminous energy) efficiency of MG@hm-$C(CN)_3$ indicates the best HER activity. (The details of calculation methods can see Supplementary Methods).

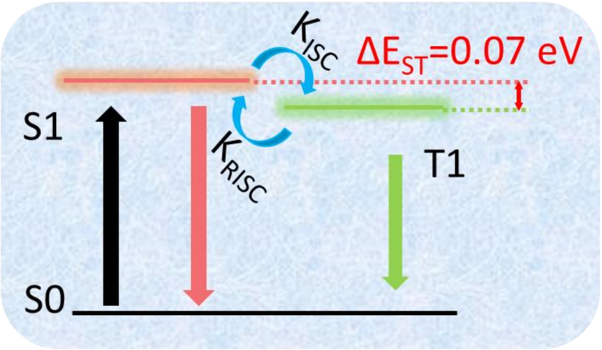


**Supplementary Figure 28. Energy diagram for singlet-triplet conversion.**

The singlet-triplet conversion is intuitively demonstrated via an energy diagram in Supplementary Figure 28. The activation energy of the ISC ($\Delta E_{ST}$) is about 0.07 eV, which is proportional to the exchange energy between the singlet and triplet energy level. The reverse ISC rate constant ($k_{RISC}$) can be estimated from the experimentally observable rate constants and the photoluminescence quantum efficiencies of the prompt and delayed components using the following equation $k_{RISC} = \frac{k_p k_d \varphi_d}{k_{ISC}\varphi_p}$, where $k_p$ = 3.16×10$^6$/s and $k_d$ = 7.46×10$^3$/s are the constants of the prompt and the delayed fluorescence components, respectively. $k_{ISC}$=1.86×10$^6$/s is the ISC rate constant from singlet to triplet states, and $\phi_p$ = 3.6% and $\phi_d$ = 5.9% are the photoluminescence quantum efficiencies of the prompt and the delayed components. The reverse ISC rate constant $k_{RISC}$=1.97×10$^4$/s, is smaller than $k_{ISC}$=1.96×10$^6$/s, indicating that a larger number of excited carriers are transferred from singlet to triplet excited states.

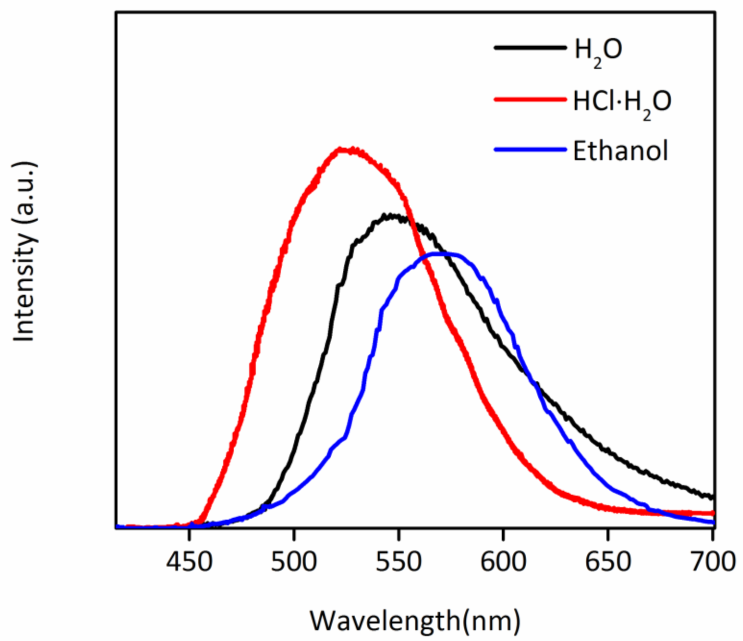


**Supplementary Figure 29. Photoluminescence spectra of R@hm-C(CN)$_3$ sample in different solvents excited by the 375 nm line of a laser**.

The PL peak position is blue-shifted from 546 nm ($H_2O$) to 524 nm (HCl $H_2O$), which can be attributed to modification of radical C site by adsorbed $H^+$. This assumption can also be confirmed by red-shift of PL peak in ethanol because the decreased amount of $H^+$ adsorbed on radical C sites leads to higher singlet-triplet conversion. These results indicate that the $H^+$ involved in HER plays an important role in singlet-triplet conversion, which is in good agreement with the conclusion of Supplementary Table 2.

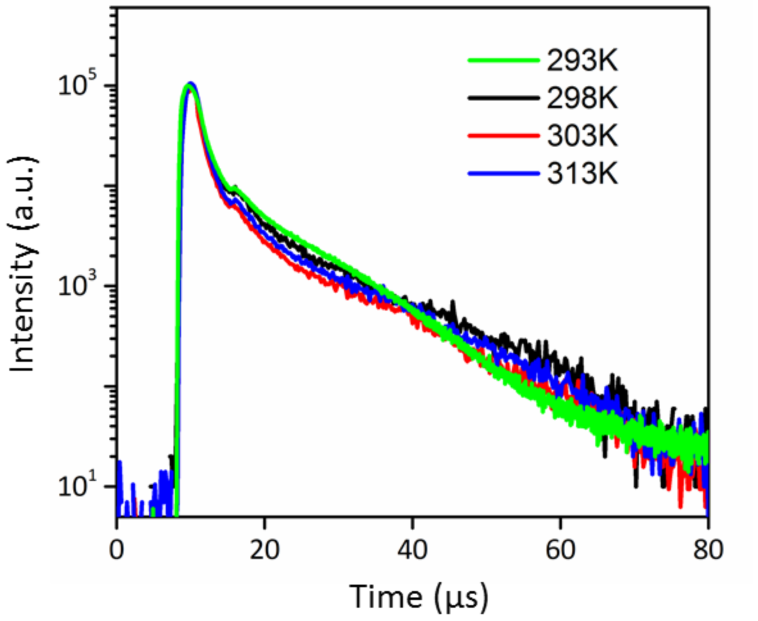


**Supplementary Figure 30. Photoluminescence decay curves acquired from the different temperatures.**

Photoluminescence decay curves of R@hm-$C(CN)_3$ at 293K, 298K, 303K and 313K (according to our experimental condition). The lifetimes of the PL emission are obtained to be $\tau_d$≈7.9 μs. The coincident photoluminescence decay curves confirm that singlet-triplet conversion cannot be affected by temperature variation in the range of 293 to 313 K.

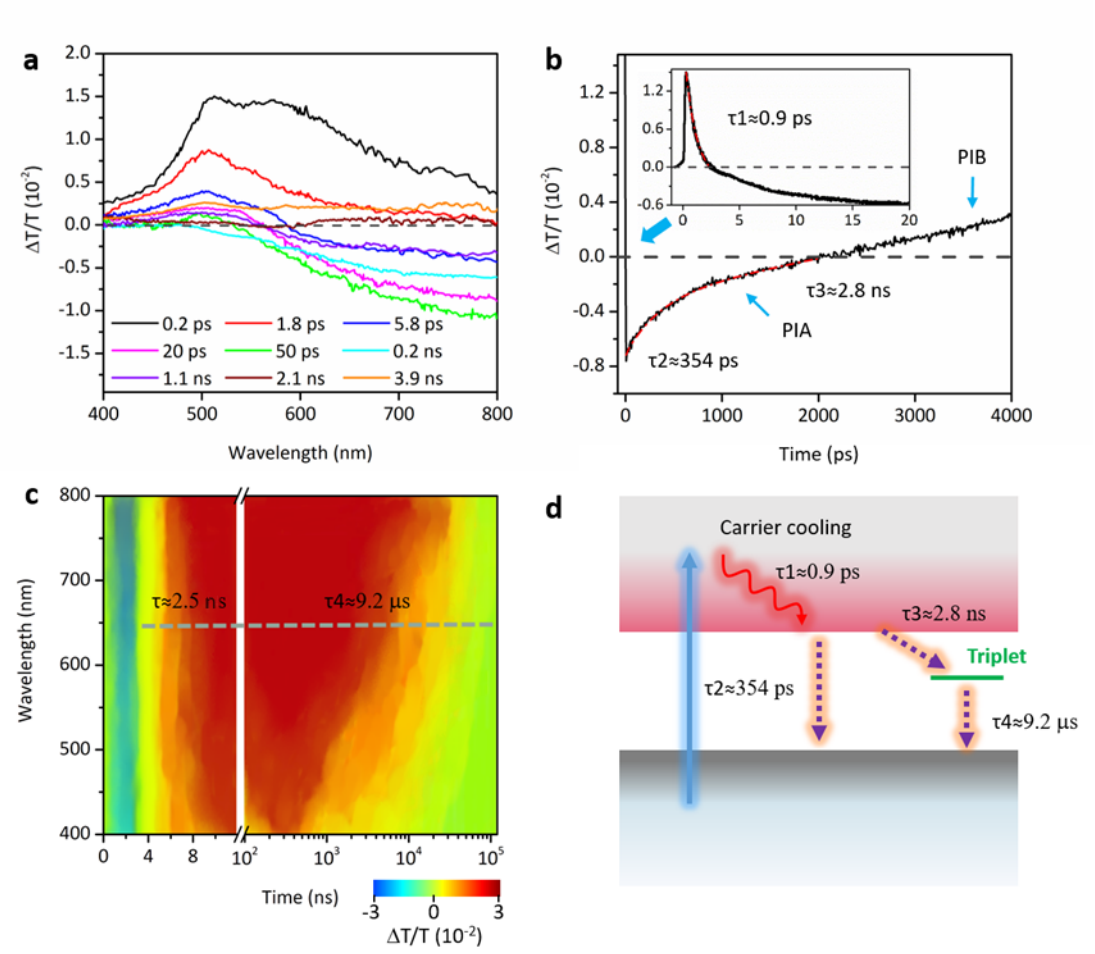


**Supplementary Figure 31. The time-resolved diffuse reflectance (TDR) spectroscopy of R@hm-C(CN)$_3$ nanosheets.**

**a** Differential transmission spectra (ΔT/T) at different time delays. **b** The curves probed at 650 nm, and the inset is the enlarged onset curve. **c** Nanosecond-resolved TA spectra in the decay range up to 100 μs. **d** Schematic model of triplet dynamics.

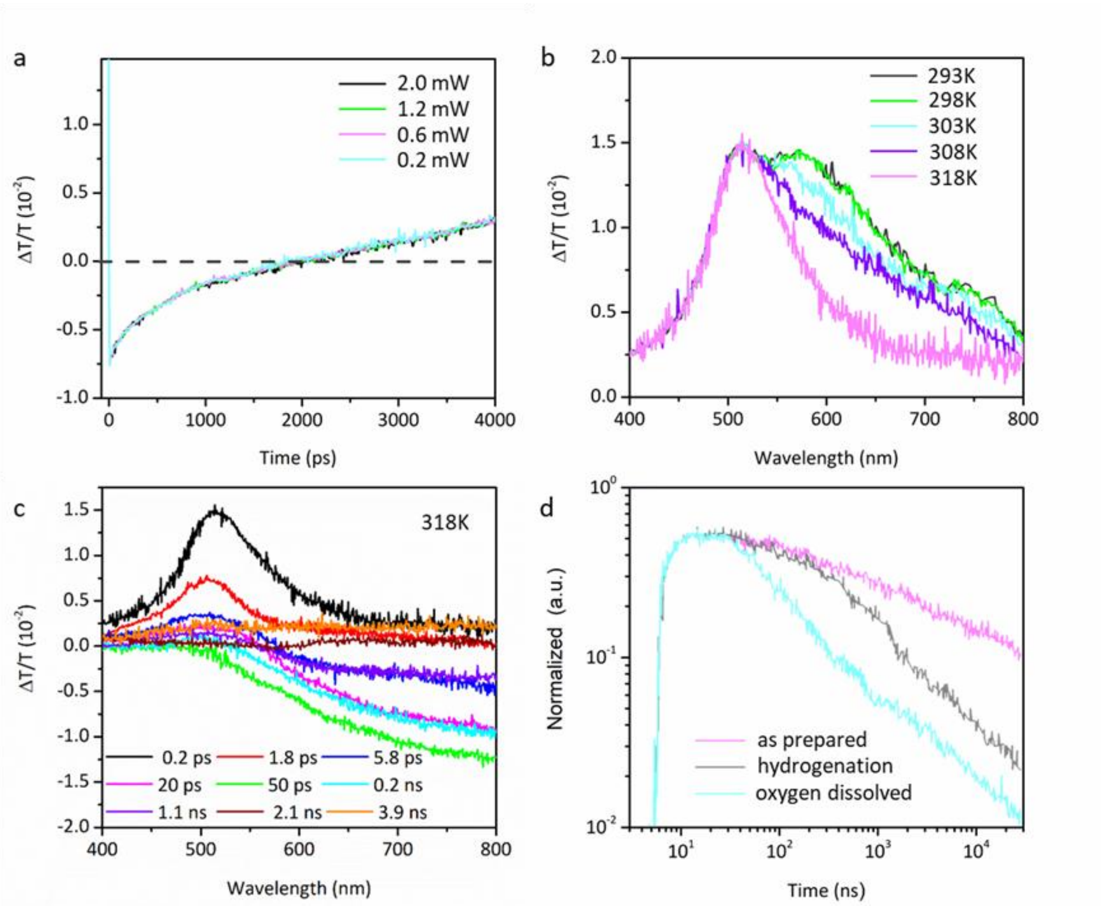


**Supplementary Figure 32. Ultrafast carrier dynamics.**

**a** The normalized curves probed at 650 nm with different pump powers. **b** The transmission spectra acquired at 0.2 ps for different measuring temperatures. **c** The differential transmission spectra at different time delays acquired at 318 K. **d** The kinetic curves probed at 650 nm recorded from R@hm-$C(CN)_3$ nanosheets before and after hydrogenation and dispersed in ethanol with dissolved oxygen.

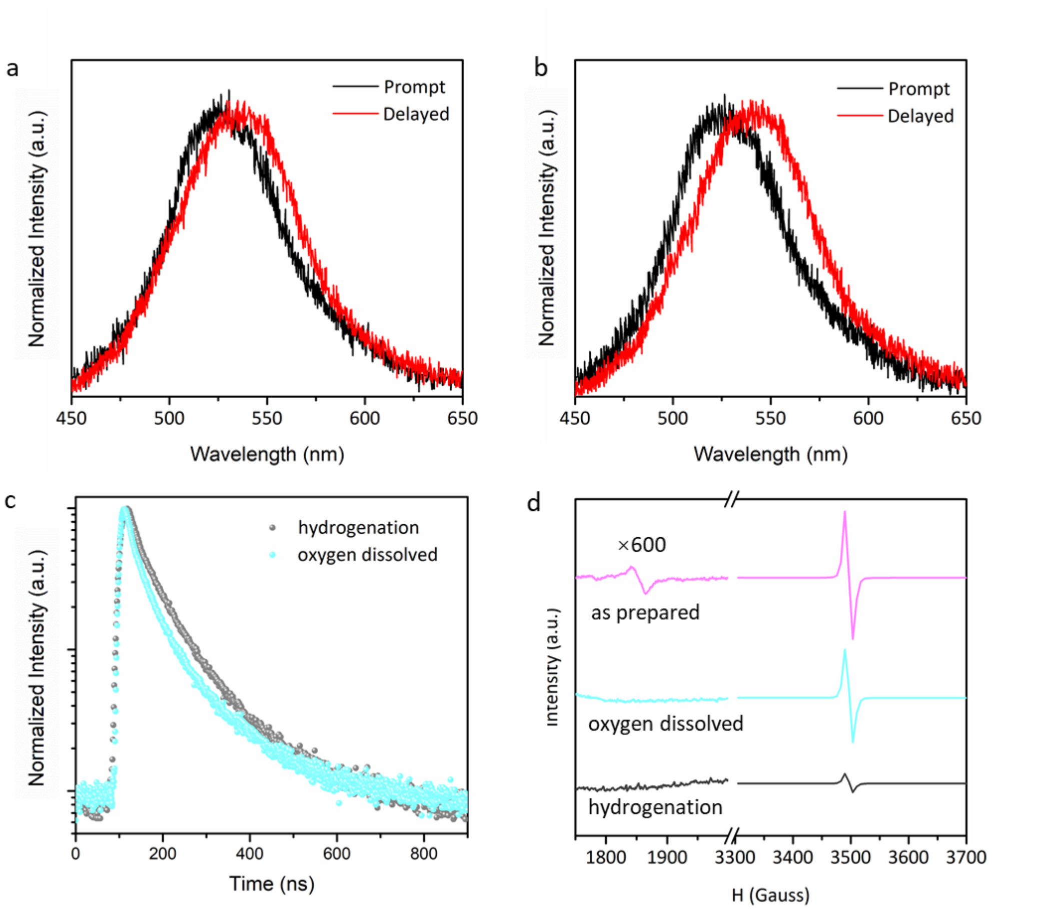


**Supplementary Figure 33.** Time-resolved photoluminescence spectra of R@hm-$C(CN)_3$ nanosheets in the presence of concentrated dissolved oxygen **a** and after hydrogenation **b**, respectively. The photoluminescence decay curves **c** and the half-field light-induced electron spin resonance (ESR) spectra **d** of R@hm-$C(CN)_3$ nanosheets in the presence of concentrated dissolved oxygen and after hydrogenation.

## Supplementary Tables

**Supplementary Table 1.** The formation energy, hydrogenated energy and reaction potential barrier for hm-$C(CN)_3$ monolayer with different defects.

| Types | Pristine | V1 | V2 | V3 |
|---|---|---|---|---|
| Formation Energy | 0.00 eV | 4.77 eV | 5.81 eV | 1.75 eV |
| Hydrogenated Energy | -2.27 eV | 1.23 eV | 1.34 eV | 0.24 eV |
| Reaction Barrier | 0.26 eV | 0.78 eV | 0.82 eV | 0.45 eV |

**Supplementary Table 2.** Physical parameters about singlet-triplet conversion for R@hm-$C(CN)_3$ nanosheets in different solvents.

| Different solvent | $\tau_p$/ratio[ns] | $\tau_d$/ratio[μs] | $\phi_p$ [%] | $\phi_d$ [%] | $K_p$ [$10^6 s^{-1}$] | $K_d$ [$10^3 s^{-1}$] | $K_{ISC}$ [$10^6 s^{-1}$] | $K_{RISC}$ [$10^4 s^{-1}$] |
|---|---|---|---|---|---|---|---|---|
| $H_2O$ (1) | 11.4 | 7.9 | 3.6 | 5.9 | 3.16 | 7.46 | 1.96 | 1.97 |
| $HCl{\cdot}H_2O$ (2) | 15.8 | 4.7 | 4.7 | 2.1 | 2.97 | 4.47 | 0.92 | 1.44 |
| Ethanol (3) | 10.9 | 8.1 | 3.5 | 6.4 | 3.21 | 7.90 | 2.08 | 2.23 |

## Supplementary Notes

**Supplementary Note 1.** The progress of hydrogenation.

The hm-C(CN)$_3$ nanosheets were placed on an alumina boat and inserted into a quartz tube center of a horizontal pipe furnace. Before heating, the system was purged with 517 standard cubic centimeter per minute (sccm) high-purity argon (Ar 99.999%) for 30 min. Then, the quartz tube was evacuated to a pressure of $1\times10^{-5}$ mbar by a mechanical pump for the duration of the reaction. After that, the furnace was heated at a heating rate of 10 ℃ min$^{-1}$ to specific temperature of 300 ℃. It was kept at this temperature for 2.5 h with a mixture of Ar (98%) and $H_2$ (2%) flow of 150 sccm. After the system cooled down to room temperature, a hydrogen-passivated product was obtained on the alumina boat. To confirm the hydrogenation process, the $^{13}$C NMR spectra of hm-C(CN)$_3$ sample before and after hydrogenation were measured and shown in Supplementary Figure 15, which also confirmed that its spin splitting is only related with radical C sites but not defects. The spin resolved photoemission spectrum of hm-C(CN)$_3$ sample after hydrogenation treatment was recorded and compared to that of before hydrogenation treatment, as shown in Supplementary Figure 16b. The spectral difference between majority and minority state disappears, in good agreement with theoretical prediction. This further confirms that the half-metallic feature does not originate from defected structures but from radical C sites, because DFT calculations show that the spin polarization induced by some defects cannot be completely eliminated, and that the difference in spin resolved photoemission spectra should remain unquenched after hydrogenation treatment.

**Supplementary Note 2.** The mechanism of scattering properties.

The scattering properties of cylindrical resonators can be described with analytical Mie theory. In Mie theory the incident, internal, and scattered fields are decomposed into a set of vector harmonics $\check{M}_m$ and $\check{N}_m$, which are indexed by their azimuthal phase dependence $e^{im\varphi}$. At normal incidence the scattered fields are purely transverse electric (TE) or transverse magnetic (TM) with excitation coefficients $a_m$ and $b_m$,

respectively:

$$a_m(x) = \frac{n_r J_m^{'}(x) J_m(n_r x) - J_m(x) J_m^{'}(n_r x)}{n_r J_m(n_r x) H_m^{'}(x) - J_m^{'}(n_r x) H_m(x)}, \quad (1)$$

$$b_m(x) = \frac{J_m^{'}(x) J_m(n_r x) - n_r J_m(x) J_m^{'}(n_r x)}{J_m(n_r x) H_m^{'}(x) - n_r J_m^{'}(n_r x) H_m(x)}. \quad (2)$$

where $J_m$ and $H_m$ are respectively Bessel and Hankel functions of the first kind and primes denote derivatives with respect to the argument. The relative refractive index $n_r$ and relative size x are given by

$$n_r = \frac{k_{\mathrm{int}}}{k_{emb}} = \frac{n_{\mathrm{int}}}{n_{emb}}; \quad x = k_{emb} r_0 = \frac{2\pi n_{emb}}{\lambda} r_0. \quad (3)$$

Where $n_{emb}$ and $n_{\mathrm{int}}$ are the refractive indexes of the embedding medium and the resonator respectively, $k$ is the wavevector, $r_0$ is the cylinder radius, and λ is the free space wavelength. The scattering and extinction properties of single resonator can be easily determined once the Mie coefficients are calculated. The cylindrical resonators make incident lights travel with multiage scattering and coherent superposition, which can efficiently increase the near infrared (NIR) absorption, as shown in Figure 3e. When hm-$C(CN)_3$ nanosheets are incorporated in the micro grid mode resonance structure, as shown in Supplementary Figures 23c-d, the photon absorption behaves in a linear way. The FDTD simulations disclose that the distribution of electric field at nanotube surface is enhanced. Moreover, when the natural light projected at the interface of the media undergoes reflection and refraction, light vector can be decomposed into two parts (as shown in Figure 4b and Supplementary Figures 23a-b). One part parallel to the incident surface is called the P wave (marked by blue arrows: $E_{1p}$), and the other part perpendicular to the incident surface is called the S wave (marked by red arrows: $E_{1s}$). In this special columnar micro grid structure, the incident natural lights are all expressed as P-wave on each plane perpendicular to the nanotube inner walls. Therefore, NIR absorption is enhanced via P wave behavior and cylindrical resonators, leading to a linear NIR absorption, as shown in Figure 3e. Besides, a hollow ring waveguide structure with AAM and air (water) as low refractive index medium is formed. Thus, all the light coupled into hm-$C(CN)_3$

experiences multiple reflections in the waveguide, which increase the effective optical path length of the light.

**Supplementary Note 3.** All parts of sunlight make contributions to the hydrogen evolution.

In order to clarify the contributions from lights of different wavelengths to the hydrogen evolution, photocatalytic $H_2$ evolution and On/Off photocurrent curves were carried out under different irradiation lights. In our measurement, 380/750 nm long-wave-pass and 380/750 nm short-wave-pass were chosen to obtain the required Vis + NIR light, pure NIR light, pure UV light and UV + Vis light, and the light intensity was calibrated to 100 mW $cm^{-2}$. Supplementary Figure 26a shows the hydrogen evolution rates with/without near infrared (NIR) or ultraviolet (UV) lights, which indicates that the hydrogen evolution rates decrease obviously in the absence of NIR or UV lights. These results strongly evidence that the entire solar energy can make the contributions to the hydrogen evolution. In addition, Supplementary Figure 26b gives the amperometric I–t curves of hm-$C(CN)_3$ photocathode at -0.2 V under the chopped light irradiation with and without NIR/UV light. Obviously, the photocurrent responds fast to each switch event in both cases. With the continuous light irradiation, hm-$C(CN)_3$ maintains reproducible photocurrent without detectable decay, indicating of its PC stability. Note that the photocurrent enhances with the addition of NIR light, whereas the bare NIR light irradiation does not yield any apparent photocurrent. Theoretically, hm-$C(CN)_3$, a semiconductor of band gap at ~550 nm, cannot be activated to generate photoelectrons under bare NIR light irradiation. This suggests that the NIR light induces a mediating effect to further promote the photoelectron output. After hm-$C(CN)_3$ sample irradiated by NIR light for 30 min, its temperature increases from 25 $^oC$ to 40 $^oC$ due to the thermal effect of NIR light, with a remarkable rise in the electric conductivity (see Figure 5a). The higher electric conductivity can promote the migration of photogenerated carriers and lead to the higher carrier mobility. Thus, the NIR-enhanced photocurrent can be

ascribed to the NIR-induced thermal effect, in good accordance with Supplementary Figure 26b. Besides, it is worth noting that bare UV light irradiation (larger than band gap $E_g$) produces a small photocurrent, but a remarkable decline in photocurrent occurs when the UV light contribution is removed. Under UV light irradiation, large numbers of photons are absorbed to generate carriers. The carriers occupied at higher energy states will relax to conduction band edge via nonradiative recombination (see analysis about time-resolved absorption spectra in Supplementary Figure 31), and then recombine with the ground states. In this recombination process, a small part of carriers will join in the hydrogen evolution reaction. Therefore, photocurrent induced by bare UV light irradiation can also be observed.

**Supplementary Note 4.** The dynamics of photoexcited carriers on different lifetime scales indicates the different physical mechanisms in R@hm-$C(CN)_3$ nanosheets.

In general, transient absorption (TA) spectrum consists of multiple components including ground-state bleaching (GSB, $\Delta T/T>0$), excited-state absorption (ESA, $\Delta T/T<0$), and stimulated emission (SE, $\Delta T/T>0$) from different excited states with their characteristic lifetime parameters Supplementary. Figure 31a shows the TA spectra recorded at different time delays with the pulse excitation at 360 nm. A photoinduced bleaching (PIB) signal appears instantaneously with the incidence of pump pulse. Following the recovery of the initial PIB signal on ps time scale, the signal of photoinduced absorption (PIA) emerges in the spectral range > 480 nm. The PIA signal peaks at a time delay of 50 ps and recovers fully on a time scale of 2.1 ns. Surprisingly, a late-stage PIB signal becomes pronounced after the recovery of the PIA signal at time delay > 2.1 ns. Subsequently, in Supplementary Figure 31b, we analyze the fs-resolved kinetic curves for the assignment of different TA components. The beginning curve is enlarged and shown in the inset, exhibiting a fast recombination dynamics on ps scale. Such a fast recombination ($\tau 1 \approx 0.9$ ps) is commonly observed during the thermalization of hot carriers whose lifetime is mainly governed by the electron-phonon (e-ph) interaction[10]. In addition, temporal evolution

of the PIA signal amplitude shows a slightly increase with longer probe time. The kinetic curve can be fitted by a biexponential decay function. The life time of the fast component is ~354 ps, comparable to the time parameter of carrier recombination. However, this fast component ($\tau2 \approx 354$ ps) of amplitude ratio less than 25% plays a much less significant role in the recovery of the photoexcited carriers. A primary channel is the much slower component with a long lifetime of 2.8 ns whose amplitude ratio is in excess of 75%. The slow dynamics may be related to the singlet-triplet intersystem crossing since the slow component extends to the buildup of a long-lived PIA signal. To further understand the relation between the PIA feature on the long time scale and singlet-triplet conversion, Supplementary Figure 31c shows the counterplots of TA signal as a function of probe wavelength and delay time measured with ns resolution. Following the recovery, the PIB signal of broadband coverage (>550 nm) gradually builds up on a time scale from several ns to 100 μs. It is reasonable to connect the long-lived feature to the triplet population as the recombination of triplet with ground state is spin forbidden. For example, the kinetics probed at 650 nm (acquired from gray line) displays that the recovery lifetime of the PIB signal is ~9.2 μs, besides the onset of bleaching ($\tau \approx 2.5$ ns). Such a long-lived component can be regarded as a solid evidence for the triplet generation. The TA feature for the long-lived triplets in the visible range manifests as the PIB, suggesting the major ESA of triplets is probably in the infrared range. The measured onset lifetime ($\tau \approx 2.5$ ns) is close to that of the major carrier relaxation channel ($\tau3 \approx 2.8$ ns) uncovered in fs-resolved TA spectroscopy, implying a high efficiency of singlet-triplet crossover. Theoretically, the rate of intersystem crossing is sensitive to energy difference between the singlet and triplet states ($\Delta E_{ST}$). Associated with transient fluorescence feature in Supplementary Figures S28-30 and Supplementary Table 2, the splitting between singlet and triplet states has been calculated to be ca. 70 meV, and the efficiency of singlet-to-triplet conversion can be roughly estimated to be ~65%. Therefore, the schematic diagram of the kinetic model describing carrier dynamics is displayed in Supplementary Figure 31d. The photoexcitation generates a hot carrier that cools down to the band edge in subps (~ 0.9 ps). A small part of the

cooled carriers may recombine with the ground state through a fast channel with lifetime of ~354 ps while the rest of carriers undergo the intersystem crossing from singlet to triplet states (~ 2.8 ns). Carriers occupying the triplet states recombine with the ground state slowly due to the spin forbidden effect (~ 9.2 μs).

**Supplementary Note 5.** The further study the generation of triplet state by TDR.

To investigate the origin about broad absorption peak from 550 to 800 nm in Supplementary Figure 31a, the pump power-dependence of this induced absorption is shown in Supplementary Figure 32a. In a considerable range of laser power, we find that the decay dynamics of the induced TA signal remains unchanged, which is obviously different from the previous reported no-geminate charge recombination[11,12]. On the contrary, this significant pump power-independent TA decay behavior is often regarded as quasi-monomolecular decay dynamics associated with neutral electron-hole pair. To further validate absorption assignment of excited-state electron-hole, we utilize electron-phonon interaction to eliminate the contribution from electron-hole pairs to broad absorption peaks at 550-800 nm. Increasing measurement temperature is a powerful method to identify the absorption spectra associated with the generation and disappearance of electron-hole pairs. Supplementary Figure 32b shows the changes in TA signal at 0.2 ps at different temperatures, which indicates that the broad absorption is quenched at higher temperature while the photoinduced bleaching signal keeps unperturbed. This temperature-dependent absorption suggests that a portion of the positive feature in the TA starting from ca. 550 to 800 nm corresponds to photogenerated electron-holes pairs. Then, we increase the temperature to 318K to exclude influence of electron-hole pairs on our assignment about long-lifetime TA components, and the induced absorption at different time delays were recorded again and shown in Supplementary Figure 32c. Interestingly, the late-stage TA signals from 20 ps to 3.9 ns is nearly unperturbed, which is quite in agreement with results observed at 298 K (Supplementary Figure 31a). This insensitivity to the temperature is an evidence of

singlet-triplet conversion occurring in our materials, which is consisted with analysis about transient fluorescence (Supplementary Figure 30) and also similar to those recently described by Chen et al[13]. In conclusion, the broad TA signals (550-700 nm) emerged at the time decays < 20 ps can be attributed to photogenerated electron-hole pairs and also can be eliminated by electron-phonon interaction, but this early stage signals cannot affect our assignment of singlet-triplet conversion at late-stage signals. The assignment of the love-lived component to triplet generation is further confirmed by an oxygen quenching measurement, i.e., by introducing oxygen into the dispersion of R@hm-$C(CN)_3$ nanosheets. The lifetime of the long-lived component is significantly shortened (Supplementary Figure 32d), indicating a strong interaction between oxygen and the excited triplet states. These results strongly support the formation of triplet state[14]. The average lifetime of the long-lived component is shortened to 1.1 μs in the presence of oxygen, suggesting that ~75% of excited triplet states may be involved in the generation of singlet oxygen. In addition, an obvious quenching behavior was also observed in R@hm-$C(CN)_3$ with hydrogenation treatment via which the half-metallic feature disappears (Supplementary Figure 16), similar to oxygen quenching curve. This confirms that the intrinsic half-metallicity plays a critical role in generation of excited triplet states. Based on the above results, we can safely describe the dynamics of photoexcited carriers in R@hm-$C(CN)_3$ nanosheets. Following the subpicosecond thermalization process to eliminate electron-hole pairs, a large portion of carriers undergo the intersystem crossing to form the triplet carriers. The long-lived triplets are affected by hydrogenation, which is critical for the hydrogen evolution reaction.

**Supplementary Note 6.** To study the generation of excited triplet states by transient photoluminescence and electron spin resonance spectra.

Considering the aforementioned transient absorption of R@hm-$C(CN)_3$ nanosheets at different pump powers (Supplementary Figure 32a) in oxygen solution and after hydrogenation (Supplementary Figure 32d), we conclude that the shifts in the PL

signals cannot originate from energy migration (either exciton diffusion or charge trapping/detrapping). To further confirm our assertion, the time-resolved PL spectra of R@hm-$C(CN)_3$ nanosheets in oxygen solution were measured again and shown in Supplementary Figure 33a. We can see that the shifted PL component is significantly reduced to 532 nm from 551 nm, because the excited triplet states are consumed for generation of singlet oxygen. In addition, similar behavior is also observed in hydrogenated samples (Supplementary Figure 33b), which indicates that singlet-triplet conversion is depressed due to disappearance of half-metallic feature. In addition, the lifetimes of the PL decay in oxygen solution and after hydrogenation are shortened from 7.9 μs to 31ns or 45 ns, respectively, as shown in Supplementary Figure 33c. This conclusion is in accordance with that of TA spectra. To further confirm the triplet generation, the half-field light-induced electron spin resonance (ESR) spectra, a convincing evidence to identify the species of generated spin triplet state, were recorded and shown in Supplementary Figure 33d. The ESR spectra under light irradiation (λ>600 nm) show very similar signals for pristine R@hm-$C(CN)_3$ sample with a Lande factor g=2.11**.** Besides this large signal which corresponds to the $\Delta M_s = \pm 1$ allowed transition, the pristine sample exhibits a weak signals (enlarged by 600 times) located at lower field values (corresponding to the $\Delta M_s = \pm 2$ forbidden transition). These can be assigned to forbidden transitions between the singlet electronic state of an exchange-coupled neighboring radical C sites and the $M_s = \pm 1$ components of the triplet state. When it is taken into account that each of these transitions is located at 2|J|/gβ to lower field of the allowed transition, exchange parameters |J| can be calculated as 0.11 $cm^{-1}$. When the oxygen is introduced into the solution of R@hm-$C(CN)_3$ nanosheets, the signals associated with the triplet ΔMs=± 1 and ΔMs= ± 2 transitions are quenched because excited triplet states are used to produce singlet oxygen. In addition, when the lone pair of electrons in R@hm-$C(CN)_3$ nanosheet are saturated by hydrogenation treatment, the ESR signals are weakened and singlet-triplet conversion also disappears. These results are well in consistence with the TA and the time-resolved PL spectra.

**Supplementary Note 7.** The structural stability of hm-$C(CN)_3$.

To distinguish whether the magnetic contribution originates from defects or from radical C1 sites in pristine hm-$C(CN)_3$, theoretical predications are provided. We investigate the relative stability of the defects by calculating the formation energy and hydrogenated energy using the following expression:

$$E_f = E_{tot}(def) + E_{def} - E_{tot}(pristine) \quad (4)$$

where $E_{tot}$(def) and $E_{tot}$(pristine) are the total energy of the (3×3) hm-$C(CN)_3$ supercells with and without defects, and $E_{def}$ is the energy of pristine C, hydrogen or nitrogen, respectively. As a general feature, the calculated results in Table S1 disclose that all defects with larger positive formation energy cannot be unintentionally introduced, and those defected structures cannot be easily deactivated by hydrogenation treatment either. This is because that the chemical bonds at defected region will be restructured to form new spin polarization, and those formed covalent bonds are too strong to unfold to react with hydrogen. On the contrary, reaction barrier calculated by climbing nudged elastic band (NEB) method[15] reveals that the radical C1 sites of pristine hm-$C(CN)_3$ can be easily deactivated by hydrogen after overcoming a 0.26 eV potential barrier, finally to achieve nonmagnetic structures with negative hydrogenation energy.

**Supplementary Note 8.** To study the singlet-triplet conversion by transient photoluminescence spectra.

In order to confirm the singlet-triplet conversion, the streak image and transient photoluminescence spectrum have been discussed in detail in Figure 6d. The physical parameters about singlet-triplet conversion for R@hm-$C(CN)_3$ in different solvents are collected and shown in Supplementary Table 2. Here, we choose HCl $H_2O$, $H_2O$ and ethanol as the solvent respectively to explore contribution of $H^+$ adsorption to singlet-triplet conversion, because they show different proton-donating abilities. Among them, HCl is the strongest proton-donor and the proton in ethanol's hydroxyl group is far less labile than in HCl or $H_2O$. The triplet excited state quantum yield $\phi_d$

in HCl $H_2O$ solvent decreases obviously, because that the radical sites are covered by $H^+$, leading to its spin polarization degeneration. In ethanol, the structural symmetry is affected slightly due to sharp drop in $H^+$ amount, which leads to larger value of $\phi_d$ . These compared results disclose that singlet-triplet conversion strongly depend on $H^+$ introduction in HER reaction.

# Supplementary Methods

## Hydrogen adsorption energy and free energy calculation

The hydrogen adsorption free energies, $\Delta G_H$, are determined in the same way as in previous studies[1]. The adsorption energy is defined as

$$\Delta E_H = E[C(CN)_3{+}H] - E[C(CN)_3] - ½\, E(H_2) \quad (5)$$

where $[C(CN)_3{+}H]$ refers to hydrogen adsorbed on the hm-$C(CN)_3$ surface, hm-$C(CN)_3$ refers to a clean hm-$C(CN)_3$ surface, and $H_2$ refers to gas phase hydrogen molecule. The hydrogen adsorption free energy is calculated at zero potential and

$$pH = 7 \text{ as } \Delta G_H = \Delta E_{H+}\ \Delta E_{ZPE}\text{-}T\Delta S \quad (6)$$

where $\Delta E_H$ is the hydrogen adsorption energy, $\Delta E_{ZPE}$ is the difference in zero point energy, T is the temperature (300 K) and ΔS is the difference in entropy between H that is adsorbed and in the gas phase, at 101325 Pa. A normal mode analysis is used to determine the vibrational frequencies of the adsorbed species, which are used to determine the zero point energy correction and the entropy. The adsorption is too strong if $\Delta G_H$ is very negative or too weak if $\Delta G_H$ is very positive.

## Calculation methods

The STH conversion effciencies were evaluated for the different photocatalysts. The total incident power ($P_{light}$) over the irradiation area (S) of 5 $cm^{-2}$ was P = I × S = 0.5 W, and the total input solar energy ($E_{light}$) for 1 h of irradiation time (t) was $E_{light}$ = P × t = 1.8 × $10^3$ J. Basing on the rate of evolved hydrogen amount ($\nu_H$), the chemical energy converted into hydrogen ($E_H$) was calculated by using an equation $E_H = \nu_H \times \Delta G \times t \times S$, where ΔG is the certain Gibbs free energy, so that the STH could be semiquantitatively estimated according to an equation：

$$\eta = \frac{E_{H_2}}{E_{light}} \times 100\% = \frac{\Delta G \times \upsilon(H_2) \times t \times S}{P_{light} \times t \times S} \times 100\% = \frac{\Delta G \times \upsilon(H_2)}{P_{light}} \times 100\%. \quad (7)$$

The quantum efficiency (QE) of different samples. The QE was calculated according to the following equation:

$$n_p = t \times S \times Q, \tag{8}$$

$$\begin{aligned} \text{QE}[\%] &= \frac{\text{number of reacted electrons}}{\text{number of incident photons}} \times 100 \\ &= \frac{\text{number of evolved H}_2 \text{ molecules} \times 2}{\text{number of incident photons}} \times 100\,. \\ &= \frac{2n_{H_2}}{n_p} \times 100 \end{aligned} \tag{9}$$

Where t is the irradiation time (s), S is the effective light irradiation area ($m^2$), and Q is the photon flux of the incident light (μmol $m^{-2}$ $s^{-1}$)

**Conduction band (CB) and valence band (VB) energy level calculation**

By subtracting the width of the He I UPS spectra (Supplementary Figure 12) from the excitation energy (21.22 eV), the ionization potential was calculated to be 6.16 eV. The $E_v$ (at 6.16 eV) and $E_c$ (conduction band energy, at 3.97 eV) of hm-C(CN)$_3$ can be converted to the electrochemical energy potential in volts (1.72 V and -0.47 V respectively) based on a reference standard where 0 V (equal to 4.44 eV when compared to the normal hydrogen electrode potential (NHE)) is plotted against $E_{vac}$ (vacuum level).

**Experimental measurement for absorption spectra**

The diffuse reflectance absorption spectra (DRS) of the samples were recorded by a UV-vis spectrophotometer (Varian Cary 5000) equipped with an integrating sphere attachment with $BaSO_4$ as a reference. In the DRS measurement, there are a large number of scattering spots in the sample so that the absorption of sample cannot be directly tested. The measurement of diffusion reflection in the sample is used to calculate the absorption according to the Kubelka-Munk (KM) theory:

$$\frac{K}{S} = \frac{(1-R_\infty)^2}{2R_\infty} = F(R_\infty). \tag{10}$$

Where the $R_\infty$ is the reflectance, K and S are the absorption coefficient and scattering coefficient respectively. F is the emission function of KM. The equation (1) can be rewritten as follows:

$$\lg F(R_\infty) = \lg K - \lg S. \tag{11}$$

If S, basically, is independent of the wavenumber, the scattering effect only influences the spectrum line along the longitudinal axis. Under this condition, $F(R_\infty)$ represents the real absorption spectrum of sample. Thus the reflectance ($R_\infty$) must be measured. The $R_\infty$ parameters of nonabsorbent materials, for example MgO and $BaSO_4$, are usually taken as references. The reflectance $R'_\infty$ can be obtained by the following equation:

$$R'_\infty = \frac{R_\infty(\text{sample})}{R_\infty(\text{reference substance})}. \tag{12}$$

During the measurement, an integrating sphere attachment with $BaSO_4$ as a reference is used to collect the diffusive light and avoid the diffusive differences caused by the light collection process. Firstly, the diffuse reflectance absorption of $BaSO_4$ powder is recorded as the background/blank sample and baseline, and then the samples were put into the integrating sphere for measurement. The background/blank ($BaSO_4$) for these measurements is automatically carried out by the UV-vis spectrophotometer.

## Supplementary References


1. Acevedo, M. C. *et al.* Efficient hydrogen evolution catalysis using ternary pyrite-type cobalt phosphosulphide. *Nat. Mater.* **14**, 1245-1251 (2015).
2. Yu, H. J. *et al.* Alkali-assisted synthesis of nitrogen deficient graphitic carbon nitride with tunable band structures for efficient visible-light-driven hydrogen evolution. *Adv. Mater.* **29**, 1605148 (2017).
3. Zhou, Z. X. *et al.* Chemical cleavage of layered carbon nitride with enhanced photoluminescent performances and photoconduction. *ACS Nano* **9**, 12480-12487 (2015).
4. Yeh, T. F., Teng, C. Y., Chen, S. J. & Teng, H. Nitrogen-doped graphene oxide quantum dots as photocatalysts for overall water-splitting under visible light illumination. *Adv. Mater.* **26**, 3297–3303 (2014).
5. Huang, M., Yan, H., Heinz, T. F. & Hone, J. Probing strain-induced electronic structure change in graphene by raman spectroscopy. *Nano. Lett.* **10**, 4074 -4079 (2010).
6. Han, Y. M. *et al.* Sub-nanometre channels embedded in two-dimensional materials. *Nat. Mater.* **17**, 129-133 (2018).
7. Zhang, C. D. *et al.* Strain distributions and their influence on electronic structures of $WSe_2$–$MoS_2$ laterally strained heterojunctions. *Nat. Nanotech.* **13**, 152-158 (2018).
8. Gao, D. Q. *et al.* Defect-related ferromagnetism in ultrathin metal-free g-$C_3N_4$ nanosheets. *Nanoscale* **6**, 2577–2581 (2014).
9. Xu, K. *et al.* Hydrogen dangling bonds induced ferromagnetism in two-dimensional metal-free graphitic-$C_3N_4$ nanosheets. *Chem. Sci.* **6**, 283–287 (2015).
10. Ge, S. *et al.* Dynamical evolution of anisotropic response in black phosphorus. *Nano. Lett.* **15**, 4650-4656 (2015).
11. Corp, K. L. & Schlenker, C. W. Ultrafast spectroscopy reveals electron-transfer cascade that improves hydrogen evolution with carbon nitride photocatalysts. *J. Am. Chem. Soc.* **139**, 7904-7912 (2017).

12. Godin, R., Wang, Y., Zwijnenburg, M. A., Tang, J. W. & Durrant, J. R. Time-resolved spectroscopic investigation of charge trapping in carbon nitrides photocatalysts for hydrogen generation. *J. Am. Chem. Soc.* **139**, 5216-5224 (2017).
13. Chen, L. *et al.* Ultrafast carrier dynamics and efficient triplet generation in black phosphorus quantum dots. *J. Phys. Chem. C* **121**, 12972-12978 (2017).
14. Zimmt, M. B., Jr, C. D. & Turro, N. J. Magnetic field effect on the intersystem crossing rate constants of biradicals measured by nanosecond transient UV absorption. *J. Am. Chem. Soc.* **107**, 6726-6727 (1985).
15. Henkelman, G. & Jonsson, H. Improved tangent estimate in the nudged elastic band method for finding minimum energy paths and saddle points. *J. Chem. Phys* **113**, 9978-9985 (2000).
