# Supplementary material for: Half-metallic carbon nitride nanosheets with micro grid mode resonance structure for efficient photocatalytic hydrogen evolution
Source: Nat Commun. 2018 Aug 22;9:3366. doi: 10.1038/s41467-018-05590-x (PMC6105617; doi:10.1038/s41467-018-05590-x)
Supplement: Supplementary file 3 — Description of Additional Supplementary Files [file 41467_2018_5590_MOESM3_ESM.pdf]

Description of Additional Supplementary Files:

Supplementary Movie 1: The progress of hydrogen generation.
